# Photoswitching FRET to monitor protein–protein interactions

**DOI:** 10.1073/pnas.1805333116

**Published:** 2018-12-31

**Authors:** Kristin H. Rainey, George H. Patterson

**Affiliations:** ^a^Section on Biophotonics, National Institute of Biomedical Imaging and Bioengineering, National Institutes of Health, Bethesda, MD 20892

**Keywords:** photoswitchable, FRET, microscopy, fluorescence, imaging

## Abstract

FRET is a powerful approach to study the interactions of fluorescent molecules, and numerous methods have been developed to measure FRET in cells. Here, we present a method based on a donor molecule’s photoswitching properties, which are slower in the presence vs. the absence of an acceptor. The technique, photoswitching FRET (psFRET), is similar to an established but underutilized method called photobleaching FRET (pbFRET), with the major difference being that the molecules are switched “off” rather than photobleached. The psFRET technique has some of the FRET imaging advantages normally attributed to fluorescence lifetime imaging microscopy (FLIM), such as monitoring only donor fluorescence. However, it can be performed on a conventional widefield microscope, requires less illumination light to photoswitch off than photobleaching, and can be photoswitched “on” again to repeat the experiment. We present data testing the validity of the psFRET approach to quantify FRET in cells and demonstrate its use in imaging protein–protein interactions and fluorescent protein-based biosensors.

## Significance

Since protein–protein interactions are extremely important in understanding normal and abnormal cell behavior, cell biologists have often relied on fluorescence techniques, such FRET, as a way to monitor interactions. FRET occurs when donor fluorescent molecules tagged to a protein of interest transfer their excited-state energy to acceptor molecules tagged to another protein of interest. The need to accurately and easily measure FRET led to our development of photoswitching FRET (psFRET). It provides advantages normally associated with advanced methods, like fluorescence lifetime imaging microscopy, with the ease and accessibility of other widely used methods, such as sensitized emission or acceptor photobleaching. The psFRET approach will greatly enhance the ability of cell biologists to utilize FRET in their studies.

Given the importance of protein–protein interactions in carrying out the normal and abnormal functions of cells, determining if interactions occur and under what conditions they occur is of paramount importance. Toward realizing this goal, researchers often utilize fluorescence imaging and FRET as an intracellular assay for protein–protein interactions in cells. FRET relies on transfer of the excited-state energy of a donor molecule to an acceptor molecule, and the distance separating the donor and acceptor must generally be within ∼10 nm for FRET to occur (1, 2). As a consequence, numerous microscopy techniques have been developed to measure FRET in cells. An extensive review of developed and theoretical FRET imaging techniques has been published (3) in addition to numerous other reviews of commonly used techniques (1, 2, 4–6). We limit our discussion to the approaches relevant to the method and analyses introduced here.

One of the most common approaches relies on measuring the increase in fluorescence from the acceptor due to the energy transfer from the donor (6). Referred to as sensitized emission approaches, these rely on excitation of the donor while monitoring the acceptor fluorescence. This signal can seldom be used unambiguously, since the sensitized emission fluorescence must be separated from the contaminating donor signal bleed through into the acceptor channel as well as fluorescence arising from direct excitation of the acceptor. However, the protocols to extract the sensitized emission signal are well established and usually require control measurements of donor alone and acceptor alone samples in conjunction with the experimental sample (7–10).

Other common approaches rely on monitoring changes in donor fluorescence as a consequence of energy transfer. After excitation, a donor molecule has numerous pathways to depopulate the excited state. The pathway most relevant for fluorescence imaging is fluorescence emission, and in the presence of a suitable acceptor, energy transfer competes with donor fluorescence emission, leading to a decrease in its signal. One of the simplest techniques to determine how much donor fluorescence is lost due to energy transfer is called acceptor photobleaching, and it only requires imaging of the donor before and after photobleaching of the acceptor (11, 12). This approach mimics measurement of the donor fluorescence signals in the presence and absence of the acceptor, which can then be used to calculate FRET efficiency. While this approach is simple and can be performed on most microscopes, artifacts due to photoconversion of the acceptor into a state that fluoresces in the donor channel (13, 14) or incomplete photobleaching can complicate analysis (1). Moreover, since the photobleached acceptors can no longer “accept” energy from the donor, acceptor photobleaching prohibits repeating the FRET measurement on the same specimen. This can be circumvented by photochromic FRET (pcFRET) using photoswitchable acceptors, which can be switched “on” and “off” (15, 16), but these analyses can also be adversely affected if the off-state absorption is not dramatically reduced (16), much like incomplete photobleaching can affect acceptor photobleaching measurements.

As described in many of the aforementioned reviews, fluorescence lifetime imaging microscopy (FLIM) of the donor molecule provides the most straightforward measure of FRET efficiency (1, 2, 17). Since in the presence of an acceptor, energy transfer competes with fluorescence emission, the result is that the donor fluorescence lifetime is decreased in direct proportion to the level of energy transfer and allows a straightforward FRET efficiency determination. While FLIM is well regarded and often considered a gold standard for FRET measurements, it requires specialized instrumentation and expertise to collect and interpret the data, making it less accessible to many researchers.

An alternative approach to determining FRET efficiencies is to use an indirect evaluation of the donor fluorescence lifetime by monitoring the donor photobleaching kinetics. For the technique of photobleaching FRET (pbFRET) (18–20), photobleaching acts as another pathway that leads to depopulation of the donor excited state. The presence of an acceptor introduces an energy transfer pathway that competes with the photobleaching pathway and decreases the rate of photobleaching compared with measurements made in the absence of an acceptor. Thus, by fitting data to exponential equations and determining the rate constants, the kinetics of photobleaching occurring on the timescale of seconds can report on changes in the fluorescence lifetime occurring on the nanosecond timescale. This method allows more straightforward data collection than FLIM–FRET, since most fluorescence microscopes can be used to monitor photobleaching. However, just as with acceptor photobleaching, the destruction of the donor molecule limits this to a single time point, and data analysis can be further complicated by photobleaching of the acceptor molecule during the donor photobleaching.

Photoswitching FRET (psFRET) introduced here follows closely the reasoning behind pbFRET. Photoswitchable fluorophores (21) undergo a reversible process in which they are photoswitched to an off state, which introduces another pathway for depopulating the donor excited state. Similar to the pbFRET example, close proximity of an acceptor to a photoswitchable donor provides an energy transfer pathway that competes with photoswitching off. This manifests as a decreased signal in the donor channel but also, slower photoswitching kinetics of the donor molecule. This behavior was reported previously for Dronpa in the presence of Atto647 (22). We have extended these studies by fitting the photoswitching decays in the presence and absence of the acceptor to determine energy transfer efficiencies. Importantly, the reversible nature of photoswitchable probes allows the donor to be photoswitched back on and followed by psFRET determinations again. Moreover, we found that the on–off contrast of Dronpa (23), the donor utilized in our experiments, offers a way to simplify sensitized emission measurements of energy transfer in at least two other data analysis approaches that do not require fitting the photoswitching decays.

To develop and test this method, we constructed a series of tandem dimers of the photoswitchable fluorescent protein, Dronpa, as the donor and the conventional red fluorescent protein, mCherry (24), as the acceptor. Additionally, by using Dronpa photoswitching kinetics as our readout, we have circumvented reliance on acceptor fluorescence and can thus mimic a major advantage of FLIM–FRET by using Ultramarine, a nonfluorescent acceptor protein (25). Here, we present tests of psFRET as an FRET imaging method, show examples monitoring histone H2B protein proximities, and describe our observations of a fluorescent protein-based caspase sensor.

## Results

FRET experiments generally consider two populations of molecules that interact to produce these signals. The desirable readout for this process is FRET efficiency, which represents the amount of excited-state energy transferred from a donor to an acceptor. Although FRET efficiency is succinctly defined as the energy transfer rate constant divided by the sum of all excited-state depopulation rate constants, it can be interpreted differently if monitoring only donor–acceptor complexes compared with an entire molecule population containing a range of interactions. For example, a small subpopulation of molecules located close together—which transfer energy very efficiently—could produce the same measured FRET efficiency as a larger subpopulation of distally located molecules transferring less efficiently. In keeping with this discussion from Zeug et al. (6), we attribute our FRET efficiency measurements to an average of the FRET efficiencies of all individual donor–acceptor complexes scaled by the fraction of donor–acceptor complexes (fractional occupancy). We include in our notation of FRET efficiency, which is usually reported simply as *E*, the fractional occupancy for either the donor (*f*_*D*_) or acceptor (*f*_*A*_) molecule population. Importantly, our measurements do not directly address fractional occupancy, but we include this notation to highlight that changes or differences in *E* (reported here as *Ef*_*D*_) can be attributed to changes in energy transfer between individual donor–acceptor complexes, changes in the levels of donor–acceptor complexes, or a combination of these factors. Thus, the FRET efficiency with respect to the donor (*Ef*_*D*_) as defined in Eq. **1** reflects this behavior by including the ratio of donor–acceptor complex concentration ([*DA*]) and total donor concentration ([*D*]):

$$Ef_{D}=\frac{E\left[DA\right]}{\left[D\right]}.$$ [1]

**Fig. 1.**
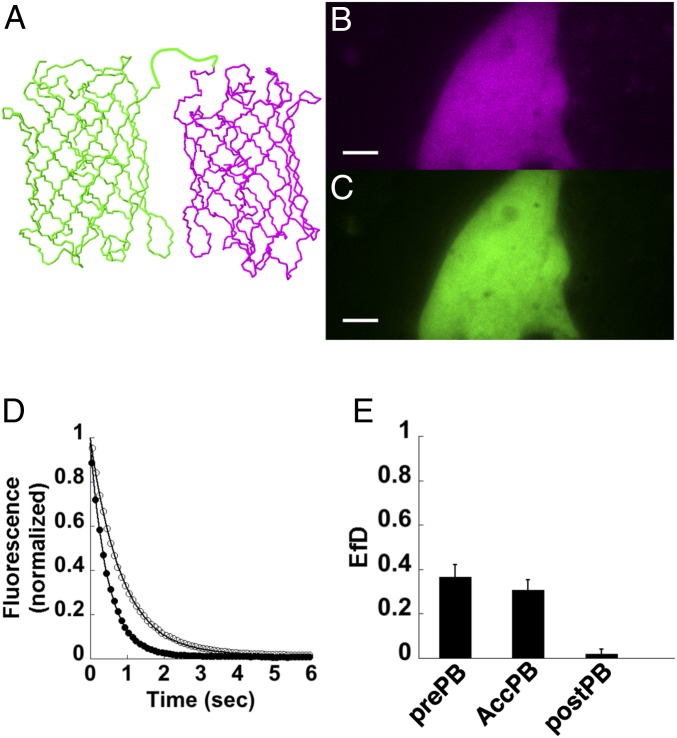
Photoswitching kinetics as a monitor for energy transfer. (*A*) The cDNA sequences for the fluorescent proteins, Dronpa (green) and mCherry (magenta), were fused to make a tandem dimer chimera with a 5-aa linker between the two proteins. (*B* and *C*) D5Ch was expressed in COS 7 cells and imaged simultaneously using a Dual-View image splitter, which shows the red channel fluorescence (*B*) on one-half of an image and the green channel fluorescence (*C*) on the other one-half. (*D*) The Dronpa signal in the green channel was measured as it photoswitched off when expressed alone (black circles) or when linked to mCherry in the D5Ch chimera (white circles). These curves were fit to a single exponential equation with offset to determine their photoswitching rate constants. (*E*) The D5Ch-expressing cells were then subjected to high-level illumination with 568-nm light to photobleach mCherry followed by a short pulse of 405-nm light to turn Dronpa back on, and then, they were subjected to another 488-nm photoswitching cycle to record a postphotobleach photoswitching decay. The D5Ch prephotobleach energy transfer efficiency (prePB) was determined from the fitted rate constants from the decays as shown in *D* using Eq. **2**. D5Ch energy transfer (AccPB) was also determined from the Dronpa fluorescence intensities measured at time 0 in the photoswitching curves before ($I_{pre}$) and after ($I_{post}$) mCherry photobleaching using Eq. **3**. The D5Ch postphotobleach energy transfer efficiency (postPB) was determined using the rate constants determined from the Dronpa fluorescence intensity decay after mCherry photobleaching. Data represent mean ± SD (*n* = 6). (Scale bars: 5 µm.)

To test psFRET as a viable alternative method for imaging energy transfer, we constructed a tandem dimer of Dronpa and mCherry using a 5-aa linker (Fig. 1*A*), expressed it in COS 7 cells (Fig. 1 *B* and *C*), and imaged it under 488-nm excitation as it photoswitched off. In parallel control experiments, Dronpa alone was expressed and imaged to determine the photoswitching rate constant in the absence of an acceptor. For these experiments, images were collected using a Dual-View imager, in which the green and red emission signals were collected simultaneously on different halves of a camera sensor (Fig. 1 *B* and *C*). An image under 568-nm excitation was collected followed by a brief pulse of 405-nm light to photoswitch Dronpa to the on state; then, images were collected under 488-nm excitation at ∼50-ms intervals. The mean fluorescence intensities were determined for Dronpa–5–mCherry (D5Ch) and Dronpa alone (Fig. 1*D*). A separate series of background images was collected on areas containing no cells, and these mean intensities were subtracted from the D5Ch and Dronpa alone images. The fluorescence decays were fitted with a single exponential decay with offset equation, and the resulting rate constants were used to determine the *Ef*_*D*_ for D5Ch using Eq. **2** (*SI Appendix* has the derivation), where $k_{DAoff}$ and $k_{Doff}$ represent the rate constants in the presence and absence of the acceptor, respectively. Since the proteins in this example were only separated by a 5-aa linker, the relatively high FRET signal (Fig. 1*E*, prePB) was expected. It was noted that the fluorescence decays fit slightly better with double and triple exponential decays, but the calculated FRET efficiencies were similar (*SI Appendix*, Fig. S1); therefore, for simplicity, we opted to fit with a single exponential with offset equation. While most of our analyses were performed using an ImageJ macro designed for our Dual-View datasets, a more versatile ImageJ plugin was developed to facilitate image analysis and curve fitting (*SI Appendix*, Fig. S2):

$$Ef_{D}=1-\frac{k_{DAoff}}{k_{Doff}}.$$ [2]

To compare psFRET with another FRET imaging method, we applied acceptor photobleaching to the same D5Ch tandem dimer. For this experiment, after monitoring Dronpa photoswitching as in Fig. 1*D* and determining *Ef*_*D*_ (Fig. 1*E*, prePB), the cells were irradiated with a high level of 568-nm laser light to photobleach mCherry. The acceptor photobleaching *Ef*_*D*_ was determined using the mean intensity of the Dronpa signal in the first image immediately after photoswitching to the on state before photobleaching (*I*_*pre*_) and after photobleaching (*I*_*post*_) using Eq. **3**:

$$Ef_{D}=\frac{I_{post}-I_{pre}}{I_{post}}.$$ [3]

We found the FRET efficiency from acceptor photobleaching experiments (Fig. 1*E*, AccPB) to be comparable with the psFRET measurements. Moreover, the second Dronpa photoswitching cycle performed after mCherry photobleaching showed only a small difference in D5Ch photoswitching kinetics compared with the Dronpa control and indicated ∼2% *Ef*_*D*_ (Fig. 1*E*, postPB). By monitoring the mCherry fluorescence in the red channel using 568-nm excitation, we noted a fluorescence loss of greater than 90% during the photobleaching step. In control experiments using Dronpa alone and mCherry alone, we did not find photobleaching using 568 nm to affect the Dronpa decay kinetics, and we did not find photoconversion of mCherry into a green component, which would complicate our acceptor pbFRET analyses. These results support psFRET as a viable alternate method for imaging energy transfer.

**Fig. 2.**
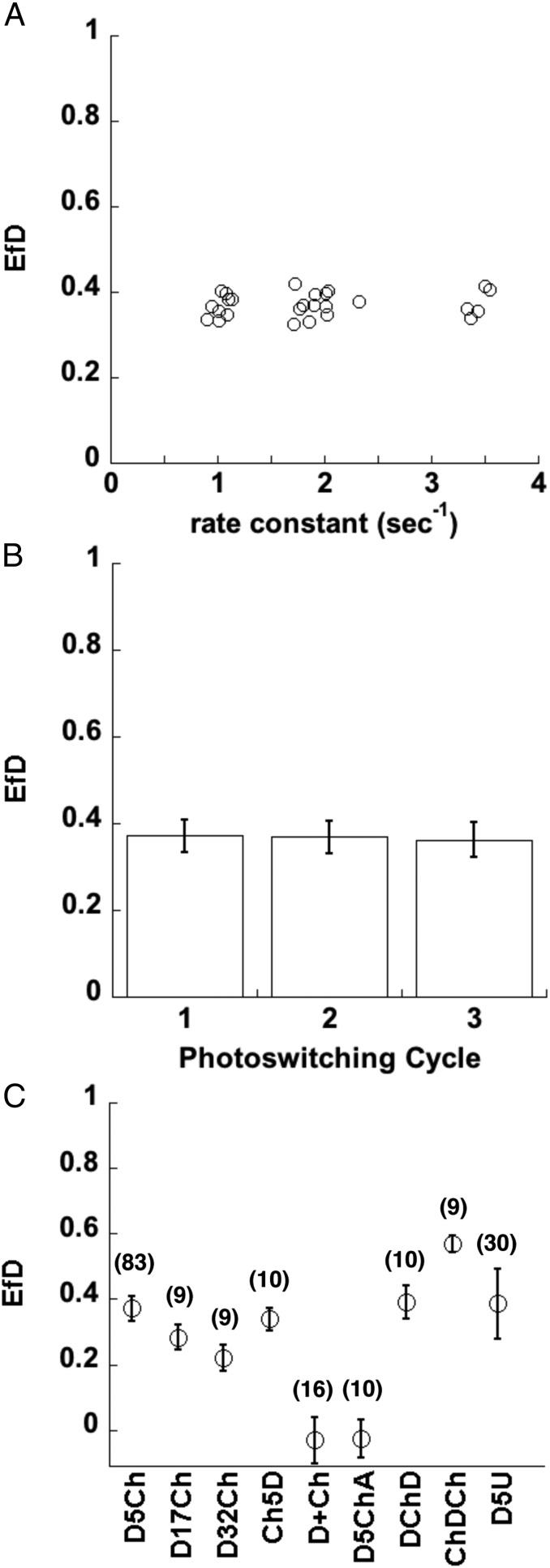
Photoswitching kinetics as a monitor of energy transfer in test chimeras. (*A*) D5Ch or Dronpa alone was expressed in COS 7 cells and photoswitched over a range of illumination intensities, which led to a range of photoswitching time constants. Each data point represents the mean *Ef*_*D*_ for D5Ch as a function of the rate constant of the Dronpa control measured over 16 datasets. (*B*) The D5Ch *Ef*_*D*_ was determined over three photoswitching cycles using Eq. **2**. Data represent mean ± SD (*n* = 83). (*C*) *Ef*_*D*_ was determined in COS 7 cells expressing D5Ch, D17Ch, D32Ch, Ch5D, Dronpa + mCherry (D+Ch), D5ChA, DChD, ChDCh, and D5U. Data represent mean ± SD, and the number of cells is indicated on the graph.

Unlike FLIM–FRET, where the lifetime is intrinsic to the molecule and largely independent of the excitation intensity, psFRET shows a dependency on the excitation intensity used to image the photoswitchable donor. Therefore, we imaged our psFRET and donor alone control experiments under the same conditions. In one such test, Dronpa was photoswitched off at several different power levels, and the *Ef*_*D*_ values of the corresponding D5Ch tandem dimer were determined. After displaying these as a function of the Dronpa alone control rate constant (Fig. 2*A*), we found a consistent FRET efficiency over a wide range of photoswitching times. Thus, we averaged the determined *Ef*_*D*_ values for the similar acceptor–donor experiments performed on multiple days of experiments. In addition, we utilized Dronpa’s photoswitching properties to measure photoswitching kinetics of the same sample over three “on–off” cycles (Fig. 2*B*).

We further tested psFRET by constructing and imaging several other Dronpa–mCherry tandem dimer variants. Increasing the linker from 5 aa to 17 or 32 aa reduced *Ef*_*D*_ (Fig. 2*C*) in keeping with the expectation that the molecules would be on average farther apart as the linker is increased (26). Reversing the sequence of expression in the tandem dimer [mCherry–5aa–Dronpa (Ch5D)] produced an *Ef*_*D*_ similar to D5Ch. As expected, expressing the Dronpa and mCherry separately resulted in no FRET given that the probable concentration range in these cells should locate them too far apart to energy transfer. We followed the logic of Koushik et al. (26) and replaced the tyrosine in the mCherry chromophore with a cysteine to disrupt the formation of an absorbing chromophore. We refer to this mCherry version of Amber as mCherryAmber (ChA), and a tandem dimer [Dronpa–5aa–mCherryAmber (D5ChA)] of Dronpa and ChA was also found to have no FRET. Additionally, we designed chimeras with varied ratios of donor to acceptor. The *Ef*_*D*_ of our Dronpa–mCherry–Dronpa (DChD) and mCherry–Dronpa–mCherry (ChDCh) showed behaviors similar to the analogous Cerulean–Venus pairings described previously (10) in that DChD produced *Ef*_*D*_ similar to D5Ch, whereas the ChDCh was much higher (Fig. 2*C*). Notably, in these chimeras, the number of donor–acceptor complexes ([DA]) is the same as the number of donors ([D]) in DChD, whereas in ChDCh, the ratio is 2:1. Therefore, the similarity of D5Ch and DChD *Ef*_*D*_ measurements as well as the large difference between DChD and ChDCh *Ef*_*D*_ determinations are consistent with Eq. **1**.

We followed these experiments with another test of psFRET’s similarity to FLIM imaging. Since psFRET measurements can be made solely by monitoring the donor molecule’s fluorescence, the acceptor molecule only needs to absorb. To test this possibility, we made a tandem dimer construct using Dronpa and Ultramarine (25), a dark absorber fluorescent protein. We linked the two proteins with the same 5-aa linker [Dronpa–5aa–Ultramarine (D5U)] and imaged it using our psFRET protocol. Given that Ultramarine has an absorption spectrum (λ_max_ = 586 nm) similar to mCherry (λ_max_ = 587 nm) and only a slightly smaller extinction coefficient (64,000 vs. 72,000), we expected and found that the *Ef*_*D*_ for D5U was similar to D5Ch (Fig. 2*C*).

**Fig. 3.**
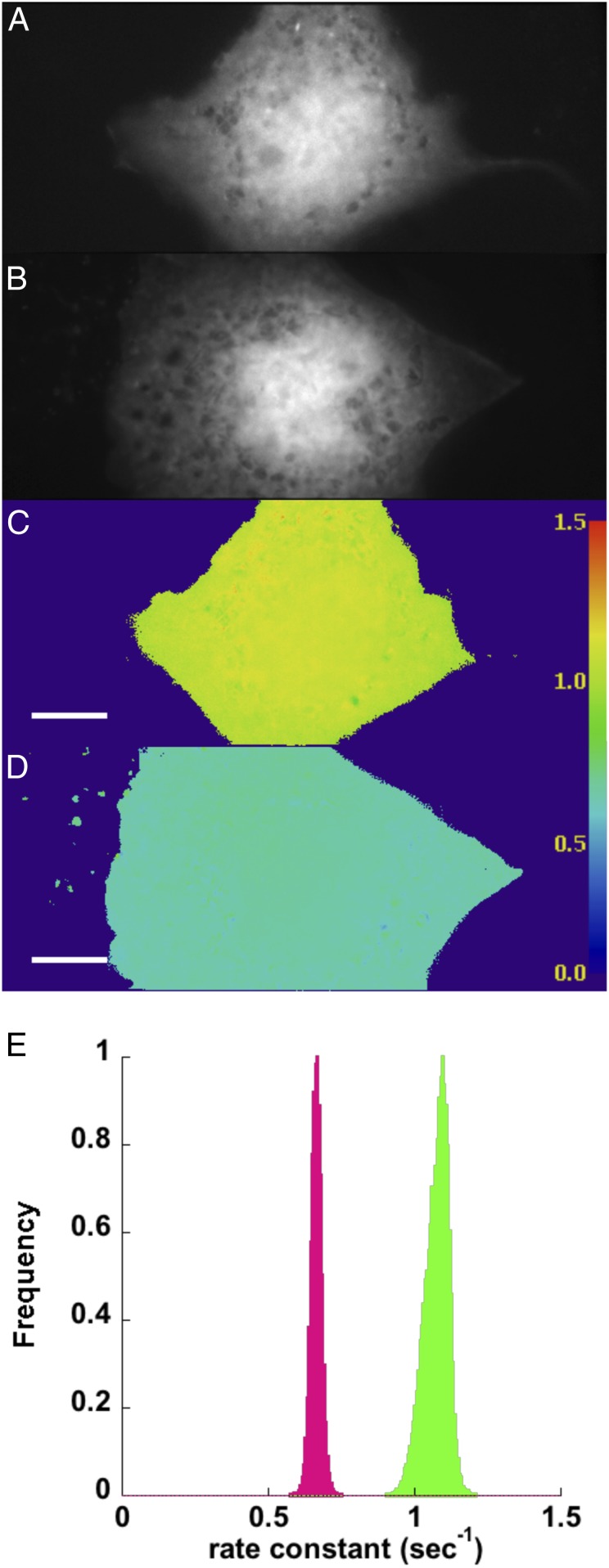
Photoswitching kinetics can be monitored on a pixel-by-pixel basis. COS 7 cells expressing Dronpa (*A*) or D5Ch (*B*) were subjected to Dronpa photoswitching. The images were thresholded to avoid attempts by the plugin to fit background. The pixel values above the threshold were extracted from the stacks at each time point and fitted to a single exponential equation with offset. The rate constant for the fit at each pixel was used to create new images of Dronpa (*C*) and D5Ch (*D*). The lookup table displayed at the right can be used to examine the rate constants (seconds^−1^) measured in different cells or regions of cells. (*E*) The pixel values from the Dronpa (green) and D5Ch (magenta) rate constant images are displayed as histograms to show the different populations. (Scale bars: 10 μm; scale is identical in all images.)

The data in Figs. 1 and 2 were produced by determining mean fluorescence intensities at specified regions of interest (ROIs) over the experimental time course and fitting the intensities with a single exponential equation. Alternatively, the photoswitching kinetics can be determined on a pixel-by-pixel basis (Fig. 3). We developed an ImageJ plugin that extracts each pixel value at every time point in the Dronpa channel (Fig. 3 *A* and *B*) and fits these to the same single exponential decay with offset equation used in our ROI method. An example of a single-pixel fit and the residuals is shown in *SI Appendix*, Fig. S2. These analyses produce images similar to FLIM images, with the exception that the resulting images contain the rate constants of the fluorescence decay in each pixel (Fig. 3 *C* and *D*) instead of fluorescence lifetimes. The plugin also outputs a reduced *χ*^2^ ($\chi_{\nu }^{2}$) image (*SI Appendix*, Fig. S3) to assay the goodness of fit for each pixel. We know from our tests (*SI Appendix*, Fig. S1) that a single exponential fit is not as precise as multiexponential fits, and with the relatively noisy single-pixel data, we do find higher $\chi_{\nu }^{2}$ values (*SI Appendix*, Fig. S3). However, spatial averaging of the pixels before fitting (analogous to the pixel binning procedure in FLIM) reduces the values in the *χ*^2^ image (*SI Appendix*, Fig. S3). We also note that the uniformity of illumination is important for comparing the rate constants across an image. By performing psFRET on a field of purified Dronpa bound to the coverslip, we found a reasonable illumination uniformity for our homebuilt machine (*SI Appendix*, Fig. S4).

**Fig. 4.**
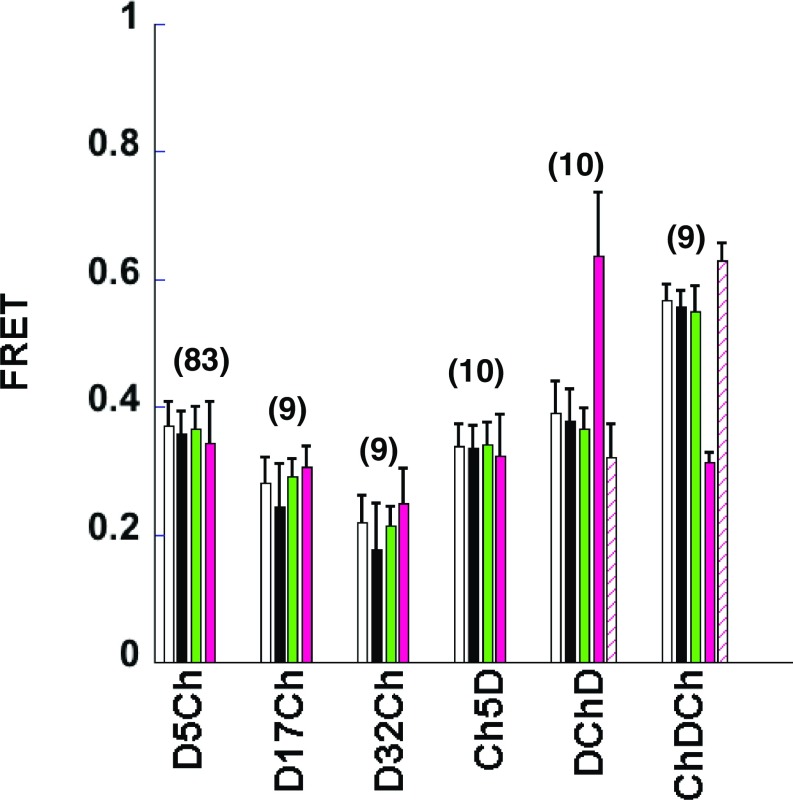
psFRET data can be analyzed using multiple approaches. COS 7 cells expressing the indicated chimera were imaged using our psFRET protocol. The white columns represent the data presented in Fig. 2*C* for comparison here. These FRET efficiencies were determined by measuring the mean pixel values in an ROI over a photoswitching cycle, fitting the decay, and using the rate constant for Dronpa alone in Eq. **2**. The black column FRET efficiencies were determined from pixel-by-pixel fits of psFRET experiments. The mean pixel values from the resulting rate constant images of the indicated chimeras were compared with the mean pixel values from the Dronpa alone rate constant images to calculate the FRET efficiencies using Eq. **2**. The green column *Ef*_*D*_ FRET efficiencies were determined using the sensitized emission signal as discussed in the text. The magenta column FRET efficiencies represent *Ef*_*A*_ determined using Eq. **11**, the sensitized emission signal, and the direct acceptor excitation as described for the green columns. Similarly, the cross-hatched magenta column FRET efficiencies represent *Ef*_*A*_ determined using Eq. **11**, in which the extinction coefficients for DChD and ChDCh were scaled *ε*_*D*_ = 125,200 mol^−1^ cm^−1^ and *ε*_*A*_ = 15,400 mol^−1^ cm^−1^ to match the number of donors or number of acceptors in the chimera, respectively. Data represent mean ± SD, and the number of cells is indicated on the graph.

Histograms of the rate constant images show the distributions associated with Dronpa alone and D5Ch (Fig. 3*E*). Using the mean pixel values from Dronpa alone and our experimental sample rate constant images in Eq. **2**, we determined FRET efficiencies (Fig. 4, black columns) and compared them with the ROI mean fitting analyses (Fig. 4, white columns). The “mean of the pixel fits” (Fig. 4, black columns) compares well with the “fit of the pixel means” (Fig. 4, white columns) and indicates that this is not only a viable alternate psFRET analysis but can perhaps make discriminating FRET differences within an ROI more straightforward.

While most of the data presented thus far relied on fitting the photoswitching decays, we note that these same datasets contain sufficient information to determine FRET efficiency by one of several sensitized emission methods, in which three images [donor excited donor emission (*I*_*DD*_), acceptor excited acceptor emission (*I*_*AA*_), and donor excited acceptor emission (*I*_*DA*_ or *FRET channel*)] are collected for every FRET measurement. Given their minimal photoswitching behaviors during imaging, conventional fluorescent proteins are generally better suited for making sensitized emission measurements. Moreover, the methods for quantitatively separating FRET form the cross-talk signals, which are well documented (7–10). However, our approach using Dronpa–mCherry FRET pairing takes advantage of the unique characteristics of using a photoswitchable fluorescent protein as the donor molecule in a sensitized emission experiment.

For any sensitized emission measurement, the fluorescence signal derived from energy transfer (*F*_*c*_) in the image *I*_*DA*_ must be separated from contaminating signals, such as the donor fluorescence bleeding into the acceptor channel and the directly excited acceptor emission. After these components are identified, simply subtracting the cross-talks will reveal the sensitized emission signal (*F*_*c*_):

$$F_{C}=I_{DA}-\text{direct acceptor excitation}-\text{donor bleed through}.$$ [4]

To remove direct excitation of the acceptor, normally a cross-talk factor (*a = I*_*DA*_*/I*_*AA*_) (*SI Appendix*) must be determined from imaging acceptor alone samples. In our case, the use of the photoswitchable fluorescent protein, Dronpa, as the donor allows for a slight modification of this equation, since it can be imaged in both on and off states. Taking advantage of this inherent property allows for a straightforward accounting of the direct acceptor cross-talk signal, eliminating the need for additional sample imaging. When off, Dronpa fluorescence is decreased 50–100 times compared with the on state, which results in donor cross-talk and energy transfer also being 50–100 times less. Therefore, the signal in *I*_*DA*_ when Dronpa is switched off approximates the direct excitation of the acceptor, mCherry. Using this reasoning, we modify Eq. **4**:

$$F_{C\;on}=I_{DA\;on}-I_{DA\;off}-\text{donor bleed through,}$$ [5]

where *F*_*c*__*on*_ is the FRET signal when Dronpa is in the on state and *I*_*DA*_ _*on*_ and *I*_*DA*_ _*off*_ are the on- and off-state fluorescence signals, respectively, in the FRET channel.

The donor bleed through is relatively straightforward to determine by imaging the donor alone. Since it is required for the psFRET protocol described earlier anyway, we measure the donor signal in both the donor channel (*I*_*DD*_) and FRET channels (*I*_*DA*_) to determine a ratio or cross-talk factor (*d = I*_*DA*_*/I*_*DD*_) (*SI Appendix*), which can be applied to every FRET experiment. While this factor is valid only for a specific donor measured on a specific microscope with a specific optical setup, it compensates for both expression level and illumination intensity. Thus, the measured donor signal in the donor channel (*I*_*DD*_ _*on*_) multiplied by the cross-talk factor (*d*) reveals the donor bleed through in *I*_*DA*_ _*on*_. Substituting this into Eq. **5** leaves only the sensitized emission signal (*F*_*c*__*on*_):

$$F_{C\;on}=I_{DA\;on}-I_{DA\;off}-I_{DD\;on}d.$$ [6]

Importantly, *F*_*c*__*on*_ only represents the fluorescence signal derived from energy transfer. To convert this into an FRET efficiency, another factor that defines the relationship between the loss in donor fluorescence due to energy transfer and *F*_*c*__*on*_ must be derived. We note that this factor has other designations in the literature (8), but here, we maintain consistency with Gordon et al. (7), Zal and Gascoigne (9), and Chen et al. (10) by referring to it as the *G* factor. Much like the donor bleed-through factor, the *G* factor is specific for each donor–acceptor pairing and optical configuration. Several methods relying on tandem dimer donor–acceptor chimeras, similar to those in our study, have been developed for determining *G* (8–10). Most applicable to the work here is that independent determination of the FRET efficiency by FLIM allows back calculation of *G* (8). We modified an equation for FRET from Chen et al. (10) by substituting *F*_*c*__*on*_, *Ef*_*D*_, and *I*_*DD*_ _*on*_ for *F*_*c*_, *E*, and *I*_*d*_, respectively (Eq. **7**). Rearrangement into Eq. **8** and substitution of *F*_*c*__*on*_, *Ef*_*D*_, and *I*_*DD*_ _*on*_ values from D5Ch psFRET measurements allowed us to calculate *G*:

$$Ef_{D}=\frac{F_{C\;on}/G}{I_{DD\;on}+\;F_{C\;on}/G}$$ [7]

$$G=\frac{F_{C\;on}\left(1-Ef_{D}\right)}{Ef_{D}\times I_{DD\;on}}.$$ [8]

Applying the *G* factor determined from our D5Ch chimera data, we calculated the *Ef*_*D*_ for all chimeras in Fig. 4 using sensitized emission. To do so, we determined *F*_*c*__*on*_ as described above, measured the donor channel fluorescence (*I*_*DD*_ _*on*_) before photoswitching, and substituted these into Eq. **7**. Since the *G* factor was determined using D5Ch, the sensitized emission-determined *Ef*_*D*_ for D5Ch was expected to match well with the psFRET determination (Fig. 4, green columns). However, we also found that sensitized emission analyses of the other chimeras matched well with their psFRET analyses (Fig. 4, green columns). These indicated that, after a *G* factor was determined, a strict reliance on fitting the photoswitching curves was not necessary to determine FRET efficiencies from our psFRET data.

Last, we present an approach using the same datasets to measure the FRET efficiency, *Ef*_*A*_, which describes FRET from the acceptor perspective (Eq. **9**):

$$Ef_{A}=\;\frac{E\left[DA\right]}{\left[A\right]}.$$ [9]

Although *Ef*_*A*_ is determined with a few FRET approaches (8), most experiments do not report it. However, since the interactions and behaviors of both donor and acceptor populations are likely of interest, this information may be beneficial. Our approach to *Ef*_*A*_ relies on an equation discussed by Lakowicz (27) and elsewhere (6, 28) to determine this readout of FRET efficiency:

$$Ef_{A}=\left(\frac{\varepsilon_{A}\left(\lambda_{D}^{ex}\right)}{\varepsilon_{D}\left(\lambda_{D}^{ex}\right)}\right)\left(\frac{F_{AD}\left(\lambda_{A}^{em}\right)-F_{A}\left(\lambda_{A}^{em}\right)}{F_{A}\left(\lambda_{A}^{em}\right)}\right).$$ [10]

Here, $\varepsilon_{A}\left(\lambda_{D}^{ex}\right)$ and $\varepsilon_{D}\left(\lambda_{D}^{ex}\right)$ are the relative excitabilities and represent the extinction coefficients of the acceptor and donor, respectively, at the donor excitation wavelength. The term $F_{AD}\left(\lambda_{A}^{em}\right)-F_{A}\left(\lambda_{A}^{em}\right)$ represents the fluorescence in the FRET channel in the presence (*F*_*AD*_) and absence (*F*_*A*_) of the donor molecule. This equation normally cannot be used directly, as measuring the FRET channel fluorescence in the absence of the donor is not generally possible. However, as previously discussed, Dronpa switches off with good contrast, providing a close approximation of FRET channel fluorescence in the absence of the donor. The term $F_{AD}\left(\lambda_{A}^{em}\right)-F_{A}\left(\lambda_{A}^{em}\right)$ is effectively the same as *F*_*c*__*on*_, which we determined as described above, and $F_{A}\left(\lambda_{A}^{em}\right)$ is the same as *I*_*DA*_ _*off*_, which we also determined for our previously described sensitized emission *Ef*_*D*_ analysis. We can now use these values and scale by the relative excitabilities to find *Ef*_*A*_:

$$Ef_{A}=\left(\frac{\varepsilon_{A}\left(\lambda_{D}^{ex}\right)}{\varepsilon_{D}\left(\lambda_{D}^{ex}\right)}\right)\left(\frac{F_{c\;on}}{I_{DA\;off}}\right).$$ [11]

For D5Ch, D17Ch, D32Ch, and Ch5D, *Ef*_*A*_ matches well with *Ef*_*D*_ (Fig. 4, magenta columns), which is expected for constructs expressing the proteins in a 1:1 ratio. However, *Ef*_*A*_ diverges from *Ef*_*D*_ measurements of DChD and ChDCh. Notably, the *Ef*_*A*_ of ChDCh is approximately the same as the 1:1 chimeras, whereas the *Ef*_*A*_ of DChD is much higher.

We considered that the disparate *Ef*_*A*_ results for the DChD and ChDCh chimeras may result from Dronpa and/or mCherry protein maturation inefficiencies. However, the consistent *Ef*_*D*_ and *Ef*_*A*_ measured for the 1:1 chimeras (Fig. 4) as well as relative Dronpa and mCherry fluorescence analyses (*SI Appendix*, Fig. S5) suggest that they may have similar folding efficiencies. Important to note here is that *Ef*_*A*_ is dependent on the ratio of the excitation wavelength extinction coefficients of mCherry and Dronpa. For the analyses in Fig. 4, we used the same extinction coefficients [$\varepsilon_{Dronpa}\left(\lambda_{Dronpa}^{488}\right)$ = 62,600 mol^−1^ cm^−1^ and $\varepsilon_{mCherry}\left(\lambda_{mCherry}^{488}\right)$ = 7,700 mol^−1^ cm^−1^] for all calculations (Fig. 4, magenta columns). However, since these data are collected on test chimeras for which we know the relative levels of Dronpa and mCherry, we can recalculate *Ef*_*A*_ after scaling their extinction coefficients accordingly. This produces DChD and ChDCh *Ef*_*A*_ values that are more consistent with the *Ef*_*D*_ measurements (Fig. 4, cross-hatched magenta columns).

**Fig. 5.**
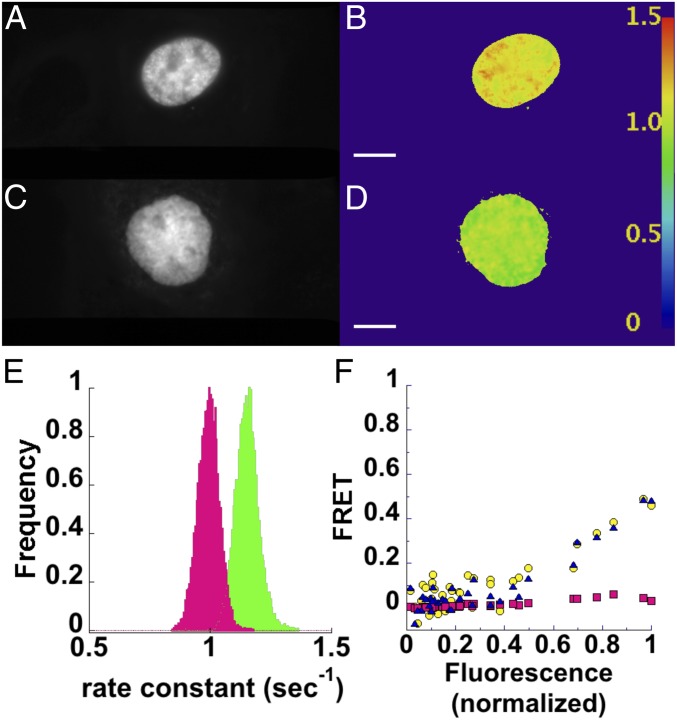
psFRET monitors FRET between histone H2B proteins in living cells. COS 7 cells expressing H2B–Dronpa (*A*) or H2B–Dronpa and H2B–mCherry (*C*) were imaged by photoswitching. Rate constant images were created from pixel-by-pixel fits of the photoswitching decays for H2B–Dronpa in the absence (*B*) or presence (*D*) of H2B–mCherry. The lookup table displayed at the right can be used to examine the rate constants (seconds^−1^) measured in different regions of these cells. (*E*) Histograms show H2B–Dronpa photoswitching rate constants in the absence (green) or presence of H2B–mCherry (magenta). (*F*) FRET efficiencies of nuclear ROIs were determined by fitting photoswitching kinetics (*Ef*_*D*_; yellow circles), from sensitized emission using Eq. **8** (*Ef*_*D*_; blue triangles), and from sensitized emission using Eq. **11** (*Ef*_*A*_; magenta squares). These are shown as a function of total fluorescence from Dronpa and mCherry normalized to the highest signal. (Scale bars: 10 µm; scale is identical in all images.)

Encouraged by the results from our experiments using tandem dimer constructs, we proceeded to test psFRET using H2B-tagged fluorescent proteins per previous FLIM–FRET studies (29). Our expression of these constructs showed nuclear localization of H2B–Dronpa (Fig. 5 *A*–*D*) as well as H2B–mCherry, both of which remained associated with chromosomes during mitosis (*SI Appendix*, Fig. S6). This result suggested an integration of these chimeras into chromatin and their potential use in studying histone protein proximities. Rate constants calculated with our pixel-by-pixel plugin (Fig. 5 *B* and *D*) showed a decrease (Fig. 5*E*) for cells expressing H2B–Dronpa and H2B–mCherry (Fig. 5*E*, magenta) compared with cells expressing H2B–Dronpa alone (Fig. 5*E*, green). Here, it is important to note that the proper FRET calculation requires the photoswitching rate constant determined in the presence of the acceptor to be compared with the rate constant of the same Dronpa chimera in the absence of the acceptor. This stems from the possibility that Dronpa photoswitching may differ depending on the local environment and is similar to protocols used in FLIM imaging. Additional analyses of H2B–Dronpa and H2B–mCherry cells were performed on mean pixels values from ROIs encompassing the nuclei using *Ef*_*D*_ sensitized emission and our calculated *G* factor (Fig. 5*F*, blue triangles), curve fitting of the photoswitching kinetics (Fig. 5*F*, yellow circles), and our *Ef*_*A*_ method (Fig. 5*F*, magenta squares). These are displayed as a function of total fluorescence (normalized to the cell with the highest level) measured at the beginning of the photoswitching cycle. Here, we note that *Ef*_*D*_ displays a marked increase with total fluorescence signal, whereas the *Ef*_*A*_ remains low at all levels. We subsequently determined the red/green fluorescence ratios in these cells and found that they were up to 20-fold higher than in cells expressing D5Ch (*SI Appendix*, Fig. S7), which suggests that the large discrepancy between *Ef*_*D*_ and *Ef*_*A*_ can be explained by the high levels of H2B–mCherry. We followed up on these findings with an experiment in which mCherry was overexpressed in the presence of D5Ch and also found *Ef*_*A*_ FRET values to be much lower than those determined for *Ef*_*D*_ (*SI Appendix*, Fig. S8).

**Fig. 6.**
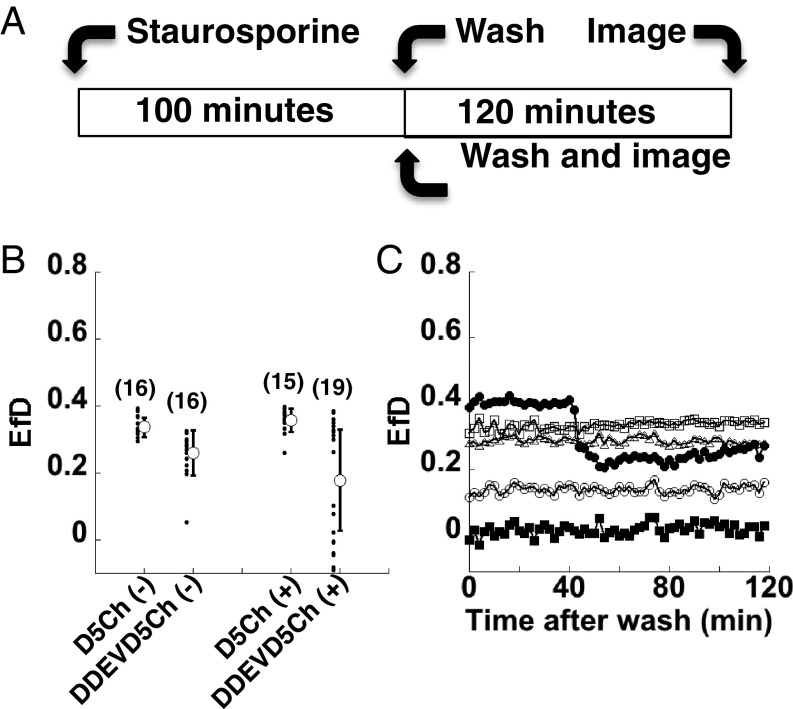
psFRET monitoring of fluorescent protein-based caspase sensor. (*A*) COS 7 cells were treated with 2 µM staurosporine for 100 min, washed, and imaged after 2 h or immediately in a time-lapse experiment over a 2-h time period. (*B*) COS 7 cells expressing the indicated chimera were untreated (−) or treated (+) with 2 µM staurosporine for 100 min and imaged using our psFRET protocol 2 h after the wash step. The white circles with error bars show the mean ± SD (number of cells is shown on the graph), and the smaller black circles indicate the FRET efficiency measured from individual cells. (*C*) Time-lapse experiments were performed on COS 7 cells expressing D-DEVD-Ch. Datasets represent FRET measurements from individual cells.

We extended our psFRET tests to a fluorescent protein-based biosensor. Here, we tested a caspase-3 sensor, referred to as DEVD, in which Dronpa and mCherry were linked with a caspase-sensitive peptide (30). Cells expressing Dronpa alone, D5Ch, or D-DEVD-Ch were treated with 2 µM staurosporine or vehicle for 100 min, rinsed with Hanks buffer, and imaged 2 h later using our psFRET photoswitching protocol (Fig. 6*A*). After determining the photoswitching rate constants, FRET efficiencies for the D5Ch(−) and D-DEVD-Ch(−) control cells (no staurosporine treatment) were calculated to be 0.337 ± 0.028 and 0.260 ± 0.068, respectively (Fig. 6*B*). We attribute this difference to the linker length (5 compared with 18 aa), much like the difference between D5Ch and D17Ch shown in Figs. 2*C* and 4. For cells treated with staurosporine, D5Ch(+) FRET was 0.359 ± 0.035, similar to no treatment, whereas the FRET for D-DEVD-Ch(+) decreased to 0.173 ± 0.182, indicating some cleavage of the caspase-3 sensor peptide. However, the observed large SD suggested heterogeneous cellular behaviors in response to the staurosporine treatment. Plotting all of the FRET efficiencies from these experiments (Fig. 6*B*, black circles) revealed two populations: one with high FRET and one with low FRET in the D-DEVD-Ch(+) cells. Additional analysis with our pixel-by-pixel fitting plugin provided additional verification of the heterogeneous response of the caspase-3 sensor in these cells (*SI Appendix*, Fig. S9).

From here, we monitored FRET efficiency over time poststaurosporine treatment. We collected psFRET cycles at 2-min intervals, and for each cycle, we determined the photoswitching rate constants for Dronpa and D-DEVD-Ch and FRET efficiency. These data (Fig. 6*C*) showed heterogeneous responses similar to the experiments performed in Fig. 6*B*. We interpreted these experiments as indicating that caspase-3 was not activated in the cells that maintained high FRET values, that it was activated before the start of our experiment in the cells with the low FRET values, or that caspase-3 was activated during a subset of our time-lapse experiments (Fig. 6*C*, black circles).

A comprehensive analysis of the data from these studies led to observations regarding Dronpa photoswitching kinetics. Control experiments performed on Dronpa and D5Ch under staurosporine treatment (*SI Appendix*, Fig. S10) showed a notable decrease in the D5Ch FRET efficiency over the experiment, which is inconsistent with our findings in Fig. 6*B*. Additional analyses showed increases in the photoswitching rate constants for both Dronpa and D5Ch over the course of 60 photoswitching cycles (120 min), and linear fits to these indicate similar slopes. Since this occurs with Dronpa alone, this suggested to us that this was an unreported Dronpa behavior rather than a consequence of the staurosporine treatment.

In efforts to better understand this behavior, we imaged Dronpa and D5Ch in similar experiments without staurosporine treatment. These again showed linear increases in the photoswitching rate constants of both Dronpa and D5Ch and a subsequent linear decrease in FRET efficiency for D5Ch (*SI Appendix*, Fig. S10). While the D5Ch rate constants were lower than Dronpa, both increased with approximately the same slope over the course of the experiment (*SI Appendix*, Fig. S10), which indicates that this linear increase was independent of Dronpa’s attachment to an FRET partner. Our observations also indicate that Dronpa and mCherry are both photostable enough to undergo numerous photoswitching cycles (*SI Appendix*, Fig. S11), suggesting that this phenomenon does not result from photofatigue or photobleaching of the donor or acceptor. We tested the cycle dependence on Dronpa photoswitching in the context of other fusions, such as Ch5D, H2B–Dronpa, Mito–Dronpa, and Vimentin–Dronpa, and found similar results (*SI Appendix*, Fig. S12). We tested Dronpa kinetics under different illumination powers and found them to produce similar slopes (*SI Appendix*, Fig. S13), further supporting a cycle-dependent phenomenon. We followed this by testing some of the available photoswitchable fluorescent proteins under the same illumination powers (*SI Appendix*, Fig. S13). Some photoswitch much faster than Dronpa under the same illumination levels, and the cycle-dependent changes in rate constant are less apparent for the proteins photoswitching much faster. For instance, SkylanS photoswitches with a rate constant closest to Dronpa and shows a similar cycle-dependent behavior. If we reduce the excitation power to better match the photoswitching rate constants to those of Dronpa, we find that SkylanNS also displays the cycle dependence (*SI Appendix*, Fig. S13). However, the rate constants for rsEGFP and rsEGFP2 do not show this apparent dependence on the cycle number.

The presence of this phenomenon in Dronpa photoswitching complicates time-lapse psFRET analysis. However, our tests suggest that the artifact’s impact can be decreased by photoswitching faster or when applicable, using a different photoswitchable fluorescent protein. However, the unexpected consistency of this behavior in both the Dronpa control D5Ch and other experiments provided a straightforward way for correction. A linear fit of the Dronpa control’s photoswitching rate constant presented a slope that we then used to correct for the change in Dronpa photoswitching kinetics at each cycle (*SI Appendix*, Fig. S10). Importantly, to avoid any possible local environmental effects on this behavior, any FRET corrections must be made using data collected from the Dronpa chimera in the absence of the acceptor. Applying this correction to the photoswitching rate constants of D5Ch led to a constant FRET efficiency over 60 cycles (*SI Appendix*, Fig. S10). This same type of correction was applied to the caspase-3 sensor data, where we still observed heterogeneous staurosporine responses of the D-DEVD-Ch cells (Fig. 6*C*). However, the corrected D5Ch FRET signals remained constant independent of staurosporine treatment (*SI Appendix*, Fig. S10).

## Discussion

We have introduced an approach to imaging energy transfer based on the photoswitching kinetics of a photoswitchable fluorescent protein (PS-FP). Previous uses of PS-FPs in FRET experiments have utilized them as acceptors and monitored the increases and decreases of donor fluorescence as the PS-FP is switched off and on, respectively (16), which allows for the monitoring of dynamics and/or averaging of responses to improve signal to noise. However, when used as a donor in an FRET pairing, Dronpa displays a slower photoswitching off rate constant in the presence vs. the absence of an acceptor, and by fitting each photoswitching curve, we can calculate energy transfer from the ratio of fitted rate constants.

The reasoning behind our study followed that of previous work monitoring photobleaching kinetics of donors in the presence of acceptors (pbFRET) (18–20), with the added advantage that the reversible nature of photoswitching allows for repeating of psFRET measurements on the same specimen. Moreover, we find PS-FP photoswitching off to be much faster than photobleaching and requires less illumination to produce the decay curves. Our motivation in pursuing the psFRET approach is also similar to that from pbFRET studies (18, 19). Namely, we are trying to mimic a well-founded technique for measuring FRET in microscopy, FLIM, which unfortunately requires specialized equipment and training inaccessible to many researchers. As discussed previously (18), a major feature of pbFRET (and subsequently, psFRET) is that it can report on the change fluorescence lifetime due to FRET by imaging much slower processes (up to nine orders of magnitude slower), which can be accomplished with most fluorescence microscopes. Moreover, we showed that psFRET can be used with nonfluorescent acceptors, which mimic another important FLIM–FRET characteristic. We anticipate that this should ease the implementation of multicolor FRET experiments on the same sample.

A potential disadvantage with psFRET for some researchers is the necessity to fit the decay curves with an exponential equation. However, we showed that the data collected for psFRET can also be used in sensitized emission analysis, which simplifies removal of one of the cross-talk signals. Using a PS-FP with high contrast between the on and off states allowed us to measure the direct acceptor excitation cross-talk contribution after the Dronpa was photoswitched off. Subtracting this signal and the donor bleed-through signal from the FRET channel signal before photoswitching off reveals the fluorescence signal due to energy transfer. Of course, this value must be converted into an FRET efficiency using a *G* factor, which we determined using one of our tandem dimer constructs. Thus, researchers who are uncomfortable relying on psFRET curve fits to determine FRET efficiencies have the sensitized emission option after a *G* factor has been determined.

How well does psFRET perform in a normal experiment as opposed to our test chimeras? Our applications include Dronpa- and mCherry-tagged versions of the histone protein, H2B. Previous FLIM–FRET studies aimed at chromatin compaction have been performed using EGFP- and mCherry-tagged H2B and noted a range of FRET efficiencies from ∼0.03 to 0.12 (29). Our data show *Ef*_*D*_ within this range, but several cells display much higher levels of energy transfer. The differences may be due to more homogeneous expression levels in the stable cell used in the previous study compared with the range of expression levels resulting from transient transfections used in our studies. Based on structures of the nucleosome (31, 32) (*SI Appendix*, Fig. S14), we do not attribute these higher levels to intranucleosome FRET, since the likely positions of the fluorescent proteins would be separated by ≥8 nm. Moreover, the D5Ch chimera *Ef*_*D*_ probably represents an approximate upper FRET limit for a 1:1 ratio of interacting proteins. Data from a few of the cells in Fig. 5*F* show *Ef*_*D*_ even higher than our D5Ch measurements, an observation that we have encountered only when Dronpa is in close proximity to more than one acceptor (for example, ChDCh in Figs. 2*C* and 4). We are currently unable to determine if these FRET interactions are occurring between neighboring nucleosomes or nucleosomes brought together by higher-order chromatin folding. However, future psFRET studies of other Dronpa- and mCherry-tagged histone proteins may be able to address these questions.

Our monitoring of a caspase-3 biosensor using psFRET showed heterogeneous cellular responses to staurosporine treatment, which were readily detected. While the heterogeneity of the biosensor was surprising, our interest was more on the utility of psFRET as a readout rather than the behavior of the caspase-3 biosensor itself. However, these studies revealed a Dronpa photoswitching behavior, which is unreported to the best of our knowledge. A subtle change in its photoswitching kinetics was found to be dependent on the number of times that it undergoes an on–off cycle, and the change is probably small enough to escape notice in previous studies. However, without correction, we found that it could result in FRET calculation errors over the course of our biosensor experiments. Additionally, while we can make corrections based on Dronpa control experiments, future goals include developing Dronpa variants that do not exhibit this behavior while maintaining its many other beneficial photoswitching characteristics.

Despite some of its pitfalls, we chose to perform our studies with Dronpa for several reasons. It is well established and has been in use for many years, which should allow researchers relying on Dronpa to seamlessly integrate psFRET into their studies. It has a high on–off contrast ratio, which allows for our sensitized emission calculation on the same data. Additionally, its photoswitching kinetics are slow compared with many of the available photoswitchable fluorescent proteins. Slow photoswitching is generally considered a disadvantage for photoswitching experiments, but from our efforts in developing psFRET, we found that it eased data collection considerably, especially before we gained experience with the technique. Moreover, we knew that we could improve the acquisition time easily by increasing the illumination power or by using one of the faster photoswitching proteins. In fact, our studies on several photoswitchable fluorescent proteins (*SI Appendix*, Fig. S13) as well as Dronpa photoswitching at different power levels (*SI Appendix*, Fig. S13) suggest that this cycle-dependent photoswitching behavior can be minimized by simply photoswitching faster. The artifact will still be present, but it will be less impactful at higher photoswitching rate constants. Thus, our recommendation for long-term psFRET imaging experiments is to either increase excitation to photoswitch Dronpa faster or use one of the rsEGFP proteins, in which we could not detect this behavior. Of course, increasing the excitation power may involve tradeoffs with phototoxicity, in which case the experiment will be better served by rsEGFP or rsEGFP2. However, the reduced on–off contrast of these proteins compared with Dronpa may make our unique sensitized emission analyses more difficult.

How does the utility of psFRET fit with the plethora of FRET imaging techniques? Acceptor photobleaching remains one of the simplest ways to perform FRET, but its reliance on photodestruction of the acceptor may introduce artifacts due to photoconversion in some experiments (13, 14), and it is limited to a single time point (1). Additionally, while the use of a photoswitchable fluorescent protein as an acceptor in pcFRET circumvents this restriction (16), it does not allow for the multiple types of analyses that we can perform with psFRET data. Our reliance on photoswitchable probes limits the number of fluorophores that can be used as donors, but since the introduction of Dronpa, several variations of photoswitchable fluorescent proteins have been developed (33), improving the probability that one or more will suffice for a given application. Since psFRET has some of the major advantages of FLIM–FRET but does not require specialized training and microscopy equipment, a researcher currently using sensitized emission measurements or acceptor photobleaching methods will likely find it readily accessible. Importantly, illumination heterogeneities and photobleaching are critical factors to control in psFRET, since the photoswitching rate constant is dependent on the illumination power levels. Moreover, psFRET imaging in samples where inner filtering or scattering of the illumination light cannot be controlled could be problematic. Photoswitching may also differ in the various local environments of a sample, and therefore, the donor alone control photoswitching rate constant must be obtained from the same chimera used for the FRET analysis. This is a typical preferred control for almost any FRET technique, and therefore, it will impose no undue burden. Given the multiple steps involved, the analyses can seem less amenable than some of the other FRET methods, but two ImageJ plugins are available to accommodate researchers interested in using psFRET. Last, the ability to turn off the donor fluorescence offers some unique capabilities in removing cross-talk signals in the FRET channel and makes possible two different sensitized emission analyses on the same datasets. Thus, psFRET offers a technically straightforward approach that can be adopted by most biologists wishing to introduce or improve FRET imaging in their studies.

## Methods

### Cell Culture.

COS 7 cells (product no. CRL-1651; ATCC) were cultivated at 37 °C under 5% CO_2_ in Bioptechs Delta-T dishes (product no. 04200417B; Bioptechs) and grown in standard DMEM-HG medium (product no. 11960; Invitrogen, Life Technologies) with 2 mM Glutamax (product no. 35050; Invitrogen), 1 mM sodium pyruvate (product no. 11360; Invitrogen), and 10% (vol/vol) heat-inactivated FBS (product no. 10082; Invitrogen). Transfections were performed using XtremeGene HP (product no. 06366236001; Roche) and incubated 24–48 h before imaging.

### Plasmid Construction.

All oligonucleotide primers were synthesized by Eurofins Genomics. PCR was performed with Phusion (New England Biolabs) or Pfu Turbo (Stratagene). Restriction enzymes for all digestions were from New England Biolabs. Digested fragments were gel purified using the QIAquick Gel Extraction Kit (Qiagen). Ligation reactions were performed with T4 DNA Ligase from Invitrogen (Life Technologies) or New England Biolabs. All newly constructed plasmids had sequences verified by Eurofins Genomics. Oligonucleotide sequences and amino acid linkers are described in *SI Appendix*.

### Microscopy.

The microscope was controlled using MicroManager (34). The excitation sources used in these experiments were a 100-mW, 405-nm LaserBoxx (Oxxius), a 100-mW Sapphire 488 nm (Coherent, Inc.), and a 150-mW Sapphire 568 nm (Coherent, Inc.). The 488 and 568 laser lines were aligned and directed through an acoustooptic tunable filter (Gooch & Housego PLC) controlled using an ESIo AOTF controller (ESImaging). The 405-nm laser current was controlled using the ESIo AOTF controller (ESImaging) and was shuttered using a homebuilt device consisting of a solenoid and relay (RobotGeek) controlled using the ESIo AOTF controller (ESImaging). The 405-nm beam was aligned with the 488- and 568-nm lines, and all were directed to the microscope using a multimode optical fiber equipped with a mode scrambler (Andor Technology Corp.) and directed toward the objective using a Di03-R405/488/561/635 dichroic mirror (Semrock). Imaging was performed using either a Nikon 100× 1.4 N.A. Plan-Apo objective lens or a Nikon 40× 1.0 N.A. Oil Plan-Apo objective lens on a Nikon TE2000. Emission light was passed through a Dual-View (Photometrics) image splitter configured with a 565dcxr dichroic mirror and 525/50 and 620/60 emission filters (Chroma Technology Corp.). Detection used either an Andor EMCCD 897 (Andor Technology Corp.) or a PCO Edge 4.2 LT (PCO AG) camera. Laser power levels were measured after the objectives using a microscope slide power sensor (part no. S170C; Thorlabs, Inc.). Power levels were estimated based on the power readings and the estimated illumination spot size depending on the objective. For 488-nm photoswitching, estimated power densities ranged from ∼0.03 to ∼0.2 W/cm^2^. For mCherry photobleaching, a separate port was utilized to decrease the illumination spot size and increase the estimated 561 power intensity to ∼2.6 MW/cm^2^.

### Image Analysis.

Images were analyzed using Fiji (35, 36) in two different ways. For one type of analysis, the mean pixel value of ROIs was extracted at each time point. These were subsequently background subtracted, normalized to the time point at the start of the photoswitching cycle, and fit with a single exponential with the offset equation “*y* = *a* × *e*^(−*bx*)^ + *c*” with the ImageJ curve-fitting function. These analyses were accomplished using a set of macros written in ImageJ to automate several of the tasks. For the second approach, each pixel was treated as an ROI. The values at each time point were extracted and fitted with a single exponential with the offset equation using the ImageJ curve-fitting function. These analyses were performed using an ImageJ plugin written to perform these tasks. The outputs are images containing the initial signal (*A*_0_), the rate constant, the offset, and the *χ*^2^ at each pixel. The digital values from the camera were converted into electrons using the conversion factor provided by the manufacturer. The weighted reduced *χ*^2^ ($\chi_{\nu }^{2}$) for each fit was calculated from the variance of the fit ***s***^**2**^ and the weighted average of the individual variances at each data point $\sigma_{t}^{2}$ from $\chi_{\nu }^{2}=s^{2}/\sigma_{t}^{2}$ (37). The parameter $s^{2}$ was calculated using $s^{2}=1/n-m\sum_{t=0}^{n}w_{t}{\left(fo_{t}-fe_{t}\right)}^{2}$, where $fo_{t}$ is the observed signal at time point ***t***, $fe_{t}$ is the expected or fit value at time point ***t***, ***n*** represents the number of images collected during the photoswitching cycle, and ***m*** represents the number of parameters used in the fit. The parameter $w_{t}$ is the weighting factor at each time point and is determined from $w_{t}=\frac{1/\sigma_{t}^{2}}{\frac{1}{n}\sum_{t=0}^{n}1/\sigma_{t}^{2}}$. To calculate the $w_{t}$, the variance at each time point was estimated using the observed signal, $fo_{t}$. Thus, the equation was modified to $w_{t}=\frac{1/fo_{t}}{\frac{1}{n}\sum_{t=0}^{n}1/fo_{t}}$. Similarly, $fo_{t}$ was used as the variance estimate in calculating the weighted average variance $\sigma_{t}^{2}=\frac{1}{\frac{1}{n}\sum_{t=0}^{n}1/fo_{t}}$. The plugin also offers the capability to examine the pixel values, the fits, the fit parameters, and the fit residuals at each pixel (*SI Appendix*, Fig. S2). Example processing times for a typical experiment on two different machines are provided in *SI Appendix*, Table S1. Additional information concerning psFRET analyses can be found on ImageJ (38). Links to macros and the compiled plugins used for psFRET analyses can be downloaded from there, and the plugin source codes can be found on GitHub (39).

## Supplementary Material

Supplementary File
