# Supplementary material for: Photoswitching FRET to monitor protein–protein interactions
Source: Proc Natl Acad Sci U S A. 2018 Dec 31;116(3):864–73. doi: 10.1073/pnas.1805333116 (PMC6338835; doi:10.1073/pnas.1805333116)
Supplement: Supplementary File [file pnas.1805333116.sapp.pdf]

Supplemental Information Appendix

Photoswitching FRET to monitor protein-protein interactions

Kristin H. Rainey and George H. Patterson

Section on Biophotonics, National Institute of Biomedical Imaging and Bioengineering, National Institutes of Health, Bethesda, MD 20892

Fluorescence lifetime imaging to measure FRET

To provide context for our subsequent derivations of photoswitching kinetics use in monitoring energy transfer, here we present a short derivation of the use of fluorescence lifetime imaging as an approach to monitor FRET. To assist readers and reviewers checking and/or correcting our derivations throughout this SI Appendix, we have included almost every step (i.e. we have shown our work) and request feedback if errors or invalid assumptions are found.

Assume a pulse of light is used to excite a population of donor molecules. This will be dependent upon the intensity of the excitation light I and the excitation rate constant k_x.

$$D \underset{k_m + k_{nr} + k_{fret}}{\overset{Ik_x}{\rightleftarrows}} D^*$$

This ignores any instrument response and considers only a population of molecules transitioning from the ground state (D) to the excited state (D^*). The molecules can return to the ground state through several pathways. The non-radiative pathways including photobleaching are lumped together as k_{nr}, the fluorescence emission pathway is denoted by k_m and the energy transfer pathway is denoted by k_{fret}.

$$\frac{d[D^*]}{dt} = -(k_m + k_{nr} + k_{fret})[D^*] \tag{1}$$

Rearrange to combine $[D^*]$ on the left side.

$$\frac{d[D^*]}{[D^*]} = -(k_m + k_{nr} + k_{fret})dt \tag{2}$$

Integrate both sides and combine the constants.

$$ln[D^*] = -(k_m + k_{nr} + k_{fret})t + C \tag{3}$$

At t=0.

$$ln[D^*] = ln[D^*]_0 = C \tag{4}$$

Substitute for C in equation 3.

$$ln[D^*] = -(k_m + k_{nr} + k_{fret})t + ln[D^*]_0 \tag{5}$$

Rearrange.

$$ln[D^*] - ln[D^*]_0 = -(k_m + k_{nr} + k_{fret})t \tag{6}$$

Use the laws of natural logarithms to combine the left side.

$$ln\frac{[D^*]}{[D^*]_0} = -(k_m + k_{nr} + k_{fret})t \tag{7}$$

Use the inverse of the natural log function to remove ln from the left side.

$$[D^*] = [D^*]_0 e^{-(k_m + k_{nr} + k_{fret})t} \tag{8}$$

Fluorescence signal is the readout for these experiments and F(t) is dependent on the excited state population and the fluorescence emission rate constant.

$$F(t) = k_m[D^*] \tag{9}$$

Rearrange and substitute for $[D^*]$ in equation 8.

$$F(t) = k_m[D^*]_0 e^{-(k_m + k_{nr} + k_{fret})t} \tag{10}$$

The coefficient of t in equation 10, $k_m + k_{nr} + k_{fret}$, provides the readout for donor fluorescence lifetime (τ_{DA}) in the presence of an acceptor.

$$\tau_{DA} = \frac{1}{k_m + k_{nr} + k_{fret}} \tag{11}$$

In the absence of an acceptor, k_{fret}= 0 and does not contribute to depopulating the excited state, so the donor fluorescence lifetime (τ_D) will be

$$\tau_D = \frac{1}{k_m + k_{nr}} \tag{12}$$

If k_{fret} is nonzero, this will result in increased depopulation of the excited state and is observed as a shorter fluorescence lifetime. If energy transfer (E) is defined by

$$E = \frac{k_{fret}}{k_m + k_{nr} + k_{fret}} \tag{13}$$

and

$$k_{fret} = (k_m + k_{nr} + k_{fret}) - (k_m + k_{nr}) \tag{14}$$

then rearranging equations 11 and 12 and substitution into equation 13 yields

$$E = \frac{\frac{1}{\tau_{\mathrm{DA}}} - \frac{1}{\tau_{\mathrm{D}}}}{\frac{1}{\tau_{\mathrm{DA}}}} \tag{15}$$

Rearrange to produce the familiar equation for determining FRET efficiency from FLIM data.

$$E = 1 - \frac{\tau_{\mathrm{DA}}}{\tau_{\mathrm{D}}} \tag{16}$$

Donor photoswitching to measure FRET

Now consider a population of photoswitchable donor molecules under constant illumination. This follows the theory developed previously for photobleaching FRET[1-3].

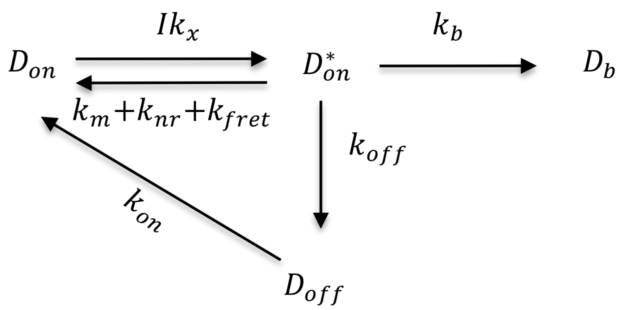

Here the various states are denoted by D_{on} for switched "ON" donors in the ground state, by D_{on}^* for switched "ON" donors in the excited state, by D_b for photobleached donors, and by D_{off} for switched "OFF" donors. The illumination intensity is indicated by *I*, the excitation rate by k_x, the fluorescence emission rate by k_m, the rate constants for the non-radiative pathways are combined as k_{nr}, the energy transfer rate constant is k_{fret}, the photobleaching rate constant is k_b, and the photoswitching "OFF" rate constant is k_{off}.

$$D_{on} \underset{k_m+k_{nr}+k_{fret}}{\overset{Ik_x}{\rightleftarrows}} D_{on}^*$$

With photoswitchable fluorescent molecules, D_{on}^* also has two other depopulation pathways.

$$D_{on}^* \xrightarrow{k_b} D_b$$

$$D_{on}^* \xrightarrow{k_{off}} D_{off}$$

It can also be photoswitched back to the "on" state by irradiation.

$$D_{off} \xrightarrow{k_{on}} D_{on}$$

For Dronpa, the donor in our experiments, photoswitching "on" requires light of a different wavelength (~400nm) than imaging and photoswitching "off" (~488nm). Therefore, this pathway is considered separate from the processes discussed below.

If we assume this system under constant illumination *I*, it can be described by the following two coupled differential equations.

$$\frac{d[D_{on}]}{dt} = -(Ik_x)[D_{on}] + (k_m + k_{nr} + k_{fret})[D_{on}^*] \tag{17}$$

$$\frac{d[D_{on}^*]}{dt} = (Ik_x)[D_{on}] - (k_m + k_{nr} + k_{fret} + k_b + k_{off})[D_{on}^*] \tag{18}$$

If we make a steady-state approximation for the excited state

$$\frac{d[D_{on}^*]}{dt} = 0 \tag{19}$$

then equation 18 rearranges to

$$(Ik_x)[D_{on}] = (k_m + k_{nr} + k_{fret} + k_b + k_{off})[D_{on}^*] \tag{20}$$

and

$$[D_{on}^*] = \frac{(Ik_x)[D_{on}]}{(k_m + k_{nr} + k_{fret} + k_b + k_{off})} \tag{21}$$

Substitute $[D_{on}^*]$ into equation 17

$$\frac{d[D_{on}]}{dt} = -(Ik_x)[D_{on}] + \frac{(Ik_x)[D_{on}](k_m + k_{nr} + k_{fret})}{(k_m + k_{nr} + k_{fret} + k_b + k_{off})} \tag{22}$$

To keep the number of terms to a manageable level, let

$$k_{all} = k_m + k_{nr} + k_{fret} + k_b + k_{off} \tag{23}$$

Expand and substitute k_{all} into equation 22

$$\frac{d[D_{on}]}{dt} = -(Ik_x)[D_{on}] + \frac{(Ik_x)[D_{on}]k_m}{k_{all}} + \frac{(Ik_x)[D_{on}]k_{nr}}{k_{all}} + \frac{(Ik_x)[D_{on}]k_{fret}}{k_{all}} \tag{24}$$

Factor $[D_{on}]Ik_x$ out of the right side.

$$\frac{d[D_{on}]}{dt} = [D_{on}]Ik_x(\frac{k_m}{k_{all}} + \frac{k_{nr}}{k_{all}} + \frac{k_{fret}}{k_{all}} - 1) \tag{25}$$

Find a common denominator

$$\frac{d[D_{on}]}{dt} = [D_{on}]Ik_x(\frac{k_m}{k_{all}} + \frac{k_{nr}}{k_{all}} + \frac{k_{fret}}{k_{all}} - \frac{k_{all}}{k_{all}}) \quad (26)$$

$$\frac{d[D_{on}]}{dt} = [D_{on}]Ik_x(\frac{k_m + k_{nr} + k_{fret} - k_{all}}{k_{all}}) \quad (27)$$

Substitute back into the k_{all} located in the numerator.

$$\frac{d[D_{on}]}{dt} = [D_{on}]Ik_x(\frac{k_m + k_{nr} + k_{fret} - (k_m + k_{nr} + k_{fret} + k_b + k_{off})}{k_{all}}) \quad (28)$$

$$\frac{d[D_{on}]}{dt} = [D_{on}]Ik_x(\frac{k_m + k_{nr} + k_{fret} - k_m - k_{nr} - k_{fret} - k_b - k_{off}}{k_{all}}) \quad (29)$$

Use arithmetic to remove several terms in the numerator.

$$\frac{d[D_{on}]}{dt} = [D_{on}]Ik_x(\frac{-k_b - k_{off}}{k_{all}}) \quad (30)$$

Rearrange to move $[D_{on}]$ to the left side and ***dt*** to the right side.

$$\frac{d[D_{on}]}{[D_{on}]} = -Ik_x(\frac{k_b + k_{off}}{k_{all}})dt \quad (31)$$

Integrate both sides and combine the constants.

$$ln[D_{on}] = -Ik_x\left(\frac{k_b + k_{off}}{k_{all}}\right)t + C \quad (32)$$

At t = 0.

$$ln[D_{on}] = ln[D_{on}]_0 = C \quad (33)$$

Substitute into equation 32.

$$ln[D_{on}] = -Ik_x\left(\frac{k_b + k_{off}}{k_{all}}\right)t + ln[D_{on}]_0 \quad (34)$$

Rearrange.

$$ln[D_{on}] - ln[D_{on}]_0 = -Ik_x\left(\frac{k_b + k_{off}}{k_{all}}\right)t \quad (35)$$

Use the laws of natural logarithms to combine the left side.

$$ln\frac{[D_{on}]}{[D_{on}]_0} = -Ik_x\left(\frac{k_b+k_{off}}{k_{all}}\right)t \tag{36}$$

Use the inverse of the natural log function to remove ln from the left side.

$$\frac{[D_{on}]}{[D_{on}]_0} = e^{-Ik_x(\frac{k_b+k_{off}}{k_{all}})t} \tag{37}$$

Rearrange.

$$[D_{on}] = [D_{on}]_0\ e^{-Ik_x(\frac{k_b+k_{off}}{k_{all}})t} \tag{38}$$

Substitute into equation 18.

$$\frac{d[D^*_{on}]}{dt} = (Ik_x)\ [D_{on}]_0\, e^{-Ik_x(\frac{k_b+k_{off}}{k_{all}})t} - (k_{all})[D^*_{on}] \tag{39}$$

Again, we assume a steady-state approximation for the excited state,

$$\frac{d[D^*_{on}]}{dt} = 0 \tag{40}$$

rearrange to move $[D^*_{on}]$ to the left side

$$(k_{all})[D^*_{on}] = (Ik_x)\ [D_{on}]_0\, e^{-Ik_x(\frac{k_b+k_{off}}{k_{all}})t} \tag{41}$$

and k_{all} back to the right side.

$$[D^*_{on}] = \frac{(Ik_x)\ [D_{on}]_0\, e^{-Ik_x(\frac{k_b+k_{off}}{k_{all}})t}}{k_{all}} \tag{42}$$

Fluorescence signal ***F(t)*** is the readout for these experiments which is dependent on the excited state population and the fluorescence emission rate constant.

$$F(t) = k_m[D^*_{on}] \tag{43}$$

Substitute for $[D^*_{on}]$ in equation 43 using equation 42.

$$F(t) = \frac{k_m(Ik_x)\ [D_{on}]_0\, e^{-Ik_x(\frac{k_b+k_{off}}{k_{all}})t}}{k_{all}} \tag{44}$$

Replace k_{all} with the rate constants.

$$F(t) = \frac{k_m(Ik_x)\,[D_{on}]_0\, e^{-Ik_x(\frac{k_b+k_{off}}{k_m+k_{nr}+k_{fret}+k_b+k_{off}})t}}{k_m+k_{nr}+k_{fret}+k_b+k_{off}} \qquad (45)$$

In the absence of an acceptor, k_{fret}= 0 and does not contribute to depopulating the excited state, but in the presence of an acceptor k_{fret} is nonzero and will result in increased depopulation of the excited state. If observed on the time scale of photobleaching or photoswitching off, this will be observed as a more slowly decaying fluorescence.

In Young et. al.[3], the authors simplified this equation by noting that k_b is orders of magnitude slower than the other terms in the denominator. And although k_{off} is faster than k_b, it is also many orders of magnitude slower than k_m, k_{nr}, or k_{fret}. For our purposes, we note that the coefficient of t in equation 45, $-Ik_x(\frac{k_b+k_{off}}{k_m+k_{nr}+k_{fret}+k_b+k_{off}})$, represents a combination of the apparent photobleaching and photoswitching rate constants. In our studies, photoswitching occurs on a much faster time scale than photobleaching, so we are effectively monitoring k_{off}. Monitoring $F(t)$ in the presence or absence of an acceptor provides the apparent k_{DAoff} and k_{Doff} values, respectively.

Once these rate constants are determined, energy transfer can be calculated by considering the definition of energy transfer.

$$E = \frac{k_{fret}}{k_m+k_{nr}+k_{fret}+k_b+k_{off}} \qquad (46)$$

Based on the coefficient $-Ik_x(\frac{k_b+k_{off}}{k_m+k_{nr}+k_{fret}+k_b+k_{off}})$ in equation 45, $k_m+k_{nr}+k_{fret}+k_b+k_{off}$ is proportional to $1/k_{DAoff}$.

$$1/k_{DAoff} \propto k_m+k_{nr}+k_{fret}+k_b+k_{off} \qquad (47)$$

and

$$1/k_{Doff} \propto k_m+k_{nr}+k_b+k_{off} \qquad (48)$$

Therefore, k_{fret} is proportional to $(1/k_{DAoff} - 1/k_{Doff})$.

$$(1/k_{DAoff} - 1/k_{Doff}) \propto k_{fret}$$

Substitution of these into equation 46 provides a calculation of energy transfer.

$$E = \frac{1/k_{DAoff} - 1/k_{Doff}}{1/k_{DAoff}} \qquad (49)$$

Rearrange to a more familiar equation.

$$E = \frac{1/k_{DAoff}}{1/k_{DAoff}} - \frac{1/k_{Doff}}{1/k_{DAoff}} \tag{50}$$

$$E = 1 - \frac{k_{DAoff}}{k_{Doff}} \tag{51}$$

Therefore, energy transfer can be determined from the fluorescence photoswitching decay in the presence (k_{DAoff}) of an acceptor compared to the absence (k_{Doff}) of an acceptor.

Sensitized emission analysis (Donor Ef_D)

Here, we consider another analysis to determine the FRET efficiency from a photoswitching FRET experiment. Although measuring the photoswitching kinetics requires imaging only in the donor channel, in practice we perform what is essentially a four-cube experiment using a Dual-View image splitter. Three of these images are the normal imaging channels associated with three-cube FRET imaging, I_{DD} (donor excitation and donor emission), I_{DA} (donor excitation and acceptor emission), and I_{AA} (acceptor excitation and acceptor emission) with the fourth being the rarely collected I_{AD} (acceptor excitation and donor emission). In general, I_{AD} is unnecessary with a proper choice of filter sets to avoid acceptor emission bleed-through into the donor emission channel, nevertheless we collect it simply due to our use of the Dual-View.

This analysis follows closely the sensitized emission approaches of Hoppe *et. al.*[4] and Chen *et. al.*[5]. We try to maintain most of the formalism of Chen *et. al.*[5] and refer readers to that work as a guide. Before we discuss our deviations from a normal three-cube experiment, we consider a typical sensitized emission FRET experiment in which I_{DD}, I_{DA}, and I_{AA} images of a sample containing a donor and acceptor are collected. In keeping with the approach of Chen *et. al.*[5], we consider all signals and crosstalk in each of these images.

$$I_{DD} = I_d + F_C\ crosstalk +\ I_a\ crosstalk \quad (52)$$

$$I_{AA} = I_a + F_C\ crosstalk +\ I_d\ crosstalk \quad (53)$$

$$I_{DA} = F_C + I_d\ crosstalk + I_a\ crosstalk \quad (54)$$

In these equations, I_d and I_a represent the directly excited donor and directly excited acceptor fluorescence signals, respectively. The crosstalk factors cannot generally be deduced from this one set of three images and require additional imaging of donor alone and acceptor alone samples. For example, the I_{DD} image (equation 52) contains the donor signal, but also may contain crosstalk from the sensitized emission FRET signal (F_c) and the directly excited acceptor (I_a). A three-cube imaging experiment on the acceptor alone sample will provide quantitative information necessary for determining these crosstalks. For each of these factors, the concentration of the acceptor or the donor will impact the level of crosstalk. Therefore, the parameters are normalized based on expression level using images I_{DD} and I_{AA}.

The crosstalk parameter, ***a***, describing the acceptor emission channel signal is derived from direct donor wavelength excitation of the acceptor can be determined from (I_{DA}) and (I_{AA}) collected on acceptor alone samples.

$$a = I_{DA}\ /\ I_{AA} \quad (55)$$

The crosstalk parameter, ***b***, describes the donor emission channel signal derived from direct excitation of the acceptor and can be determined from the images I_{DD} and I_{AA} of the acceptor alone.

$$b = I_{DD} \ / \ I_{AA} \tag{56}$$

The I_{AA} image (equation 53) contains the acceptor excited signal (I_a), but also may contain crosstalk emission from the donor in the form of directly excited donor (I_d) and sensitized emission (F_c). The crosstalk parameter, ***c***, describes donor emission channel signal derived from direct excitation by the acceptor channel and can be determined from the images I_{DD} and I_{AA} of the donor alone. Similarly, the donor concentration will impact the level of crosstalk, so the parameters are normalized to (I_{DD}), which provides the readout for the donor level.

$$c = I_{AA} \ / \ I_{DD} \tag{57}$$

The crosstalk parameter, ***d***, describes the FRET channel signal derived from direct donor wavelength excitation of the donor and bleed-through into the acceptor emission channel. It can be determined from (I_{DA}) and (I_{DD}) collected on donor alone samples.

$$d = I_{DA} \ / \ I_{DD} \tag{58}$$

The image I_{DA} (equation 54) contains the sensitized emission FRET signal (F_c) in which we are interested but also has artifact signals from the donor excited donor fluorescence (I_d) and the donor excited acceptor fluorescence (I_a). While crosstalk in I_{DD} and I_{AA} are usually small, the crosstalk in the I_{DA} image can often be substantial depending on the fluorophore pairing. With these parameters, equations 52-54 can be modified to quantitatively reflect the relative crosstalks.

$$I_{DD} = I_d + F_C \left(\frac{b}{a}\right) + \ I_a \, b \tag{59}$$

$$I_{AA} = I_a + F_C \left(\frac{c}{d}\right) + \ I_d \, c \tag{60}$$

$$I_{DA} = F_C + I_d \, d + I_a \, a \tag{61}$$

The next step in determining a FRET efficiency requires extraction of F_c from the I_{DA} image by removing the crosstalk signals. By rearranging equations 59-61, the signals for I_d, I_a, F_c are isolated.

$$I_d = I_{DD} - F_C \left(\frac{b}{a}\right) - \ I_a \, b \tag{62}$$

$$I_a = I_{AA} - F_C \left(\frac{c}{d}\right) \ - \ I_d \, c \tag{63}$$

$$F_C = I_{DA} - I_d\, d - I_a\, a \tag{64}$$

Equations 62 and 63 can be further derived to express I_d and I_a as functions of I_{DD}, I_{AA}, I_{DA} and the crosstalk parameters, but these simplify considerably if the parameters ***b*** and ***c*** are approximated as 0, which is valid for properly chosen excitation and emission filters. For example, ***b*** and ***c*** in Chen et. al [5] were found to be 0.0004 and 0.0013, respectively and in our system, we found them to be 0.00447±0.00255 and -0.00008±0.00011, respectively (mean±sem). Assuming ***b*** = 0 and ***c*** = 0, then equations 56 and 57 simplify to

$$I_d = I_{DD} \tag{65}$$

$$I_a = I_{AA} \tag{66}$$

Substituting into equation 64 provides an equation for using a normal three-cube set of images and the predetermined crosstalk parameters ***a*** and ***d*** to extract the sensitized emission signal from the I_{DA} image.

$$F_C = I_{DA} - I_{DD}\, d - I_{AA}\, a \tag{67}$$

Next, we discuss our deviations from a normal three-cube experiment for extracting F_c from the image I_{DA} in our photoswitching FRET experiments. Since the photoswitching FRET experiment requires imaging of the donor (Dronpa in our case) as it switches off, we also must measure the photoswitching kinetics of the donor in the absence of the acceptor in another sample. Therefore, we will have the sample control necessary to determine donor crosstalk into the FRET channel, which in the conventional three-cube experiment discussed earlier is the crosstalk parameter ***d***. Bear in mind that our use of the image splitter makes convenient the imaging of the donor channel (I_{DD}) and FRET (I_{DA}) simultaneously, but this is not a strict requirement. For the Dronpa alone samples measured on our system, ***d*** = 0.04253±0.00044 (mean±sem).

Here we deviate from a normal 3-cube data collection. Although we do collect image I_{AA} in our experiments and have determined ***a*** from mCherry expressing cells for some experiments, we generally do not for every experiment and therefore do not use the crosstalk parameter, ***a***, to calculate the acceptor direct excitation crosstalk. Instead, we rely on photoswitching "off" of the donor during the experiment which decreases both the donor bleed-through and sensitized emission from the I_{DA} image. The signal in the I_{DA} image at the end of a photoswitching cycle provides a good approximation of the I_a crosstalk and acceptor levels.

Consider again equation 61, which describes the components of the FRET image, but this time we consider I_{DA} at the beginning of the experiment before photoswitching off ($I_{DA\ on}$) and after the photoswitching cycle ($I_{DA\ off}$).

$$I_{DA\ on} = F_{C\ on} + I_{d\ on}\, d + I_{a\ on} \tag{68}$$

$$I_{DA\,off} = F_{C\,off} + I_{d\,off}\,d + I_{a\,off} \tag{69}$$

Before photoswitching, $I_{DA\,on}$ has the same crosstalk as a conventional three-cube experiment, so we include the crosstalk parameter ***d*** for $I_{d\,off}$. Since the donor fluorescence is decreased, it will also decrease its probability to energy transfer and $F_{c\,off}$ will also be decreased. Here we make the assumption that $F_{c\,off}$ and $F_{c\,on}$ will be proportional to donor fluorescence in the respective "off" and "on" states.

$$\frac{F_{C\,off}}{I_{d\,off}} = \frac{F_{C\,on}}{I_{d\,on}} \tag{70}$$

Rearranging this provides $F_{c\,off}$.

$$F_{C\,off} = F_{C\,on}\left(\frac{I_{d\,off}}{I_{d\,on}}\right) \tag{71}$$

Substitution into equation 69 gives the following.

$$I_{DA\,off} = F_{C\,on}\left(\frac{I_{d\,off}}{I_{d\,on}}\right) + I_{d\,off}\,d + I_{a\,off} \tag{72}$$

Rearrange equation 72.

$$I_{a\,off} = I_{DA\,off} - F_{C\,on}\left(\frac{I_{d\,off}}{I_{d\,on}}\right) - I_{d\,off}\,d \tag{73}$$

If we assume the direct excitation of the acceptor is stable.

$$I_{a\,off} = I_{a\,on} \tag{74}$$

Then equation 73 can be substituted into equation 68.

$$I_{DA\,on} = F_{C\,on} + I_{d\,on}\,d + I_{DA\,off} - F_{C\,on}\left(\frac{I_{d\,off}}{I_{d\,on}}\right) - I_{d\,off}\,d \tag{75}$$

Rearrange equation 75 and isolate $F_{c\,on}$.

$$F_{C\,on} - F_{C\,on}\left(\frac{I_{d\,off}}{I_{d\,on}}\right) = I_{DA\,on} - I_{DA\,off} - I_{d\,on}\,d + I_{d\,off}\,d \tag{76}$$

$$F_{C\,on}\left(1 - \frac{I_{d\,off}}{I_{d\,on}}\right) = I_{DA\,on} - I_{DA\,off} - I_{d\,on}\,d + I_{d\,off}\,d \tag{77}$$

$$F_{C\,on}\left(\frac{I_{d\,on}}{I_{d\,on}} - \frac{I_{d\,off}}{I_{d\,on}}\right) = I_{DA\,on} - I_{DA\,off} - I_{d\,on}\,d + I_{d\,off}\,d \quad (78)$$

$$F_{C\,on}\left(\frac{I_{d\,on} - I_{d\,off}}{I_{d\,on}}\right) = I_{DA\,on} - I_{DA\,off} - I_{d\,on}\,d + I_{d\,off}\,d \quad (79)$$

$$F_{C\,on} = \left(\frac{I_{d\,on}}{I_{d\,on} - I_{d\,off}}\right)(I_{DA\,on} - I_{DA\,off} - I_{d\,on}\,d + I_{d\,off}\,d) \quad (80)$$

If we assume $I_d = I_{DD}$ from equation 65, then we can substitute into equation 80.

$$F_{C\,on} = \left(\frac{I_{DD\,on}}{I_{DD\,on} - I_{DD\,off}}\right)(I_{DA\,on} - I_{DA\,off} - I_{DD\,on}\,d + I_{DD\,off}\,d) \quad (81)$$

After the donor is photoswitched off, $I_{DD\,off}$ is decreased. In our experiments on Dronpa alone expressing cells, the intensity in the Dronpa $I_{DD\,off}$ image is approximately 0.01371±0.00074 of the Dronpa $I_{DD\,on}$ image. Subsequently, using $\boldsymbol{d}$ = 0.04253±0.00044, the crosstalk fluorescence intensity in the post photoswitching image $I_{DD\,off}\,d$ will be approximately 0.00058 of the $I_{DD\,on}$ image. Therefore, we approximate the donor crosstalk in the post photoswitching image as zero and simplify equation 81.

$$F_{C\,on} = \left(\frac{I_{DD\,on}}{I_{DD\,on} - I_{DD\,off}}\right)(I_{DA\,on} - I_{DA\,off} - I_{DD\,on}\,d) \quad (82)$$

If a small underestimation of $F_{c\,on}$ and thus underestimation of the energy transfer is acceptable, we can further simplify equation 82 by assuming $I_{DD\,off} = 0$. As mentioned earlier, the "off" state fluorescence of Dronpa is approximately 0.01371±0.00074 of the "on" so the underestimation in $F_{c\,on}$ will be <2%. Setting $I_{DD\,off} = 0$ gives a straightforward equation to separate the sensitized emission from the crosstalk artifacts.

$$F_{C\,on} = I_{DA\,on} - I_{DA\,off} - I_{DD\,on}\,d \quad (83)$$

In this equation, we are subtracting the direct excitation of the acceptor determined when the donor is switched off ($I_{DA\,off}$) and the donor bleed-through ($I_{DD\,on}$) scaled by a predetermined crosstalk parameter ($\boldsymbol{d}$) from the FRET channel image before photoswitching ($I_{DA\,on}$).

However, just as in the three-cube experiment, the $F_{C\,on}$ value is simply the sensitized emission signal and not a FRET efficiency. As has been recounted in numerous papers [4-7] and reviews[8-10], the best option is to convert this into a FRET efficiency which

can be compared across instruments and laboratories. To do so, one must determine a ***G*** or **ϒ** factor which is specific for each imaging system and fluorophore pairing. The ***G*** factor is a ratio relating the sensitized emission signal (F_C) to the loss in donor fluorescence due to energy transfer. Again, numerous approaches have been devised to determine ***G***, but our sensitized emission analysis uses a similar approach as Hoppe *et. al.* [4] and the equation and terminology from Chen *et. al.* [5].

Chen *et. al.* [5] based their use of the ***G*** factor on work from Zal and Gascoigne [7] and relied on a determination through donor-acceptor chimeras with different length linker peptides.

$$E = \frac{F_C/G}{I_d + F_C/G} \tag{84}$$

Hoppe *et. al.* [4] relied on determining energy transfer of a single donor-acceptor chimera using FLIM-FRET. Thus, with an independent measurement of energy transfer, the ***G*** factor (**ϒ** in [4]) could be determined after removal of the crosstalk signals in the FRET channel and measuring the donor channel fluorescence of the same donor-acceptor chimera. Since we can measure FRET efficiency of one of our chimeras by fitting the photoswitching kinetics and we can isolate the sensitized emission FRET signal in a straightforward manner, we rearranged equation 84 and back calculated a **G** factor for our instrument and donor-acceptor pairing.

$$E(I_d + \frac{F_C}{G}) = F_C/G \tag{85}$$

$$EI_d = \frac{F_C}{G} - E\frac{F_C}{G} \tag{86}$$

$$EI_d = \frac{F_C}{G}(1 - E) \tag{87}$$

$$G = \frac{F_C(1 - E)}{EI_d} \tag{88}$$

Once the ***G*** factor (0.15603±0.00197) was determined for one of our donor-acceptor chimeras (D5Ch), we used it in equation 84 to determine the FRET efficiency from our measurements of the sensitized emission signals from our other chimeras and experimental samples.

Sensitized emission analysis (Acceptor Ef_A)

Next, we consider energy transfer from the perspective of the acceptor. Although energy transfer is generally considered and expressed as the loss from the donor, it can also be expressed as a measure of energy gained by the acceptor and thus provide a read-out of the fraction of acceptors in complex with the donor molecules. The sensitized emission signal ($F_{c\,on}$) described above will have the same spectrum as that of the direct acceptor excitation ($I_{a\,on}$) with the relationship dependent on the relative absorption of the donor and acceptor at the donor excitation wavelengths. Consider the following equation taken from Lakowicz[11] and discussed elsewhere[4,10,12] in which the energy transfer is described for sensitized emission of the acceptor.

$$E = \left(\frac{\varepsilon_A\,(\lambda_D^{ex})}{\varepsilon_D\,(\lambda_D^{ex})}\right)\left(\frac{F_{AD}\,(\lambda_A^{em})}{F_A\,(\lambda_A^{em})} - 1\right)\left(\frac{1}{f_D}\right) \tag{89}$$

In Lakowicz[11], f_D was used to express fractional labeling of the acceptor, whereas subsequent discussions [4,10,12] used f_A. Equation 89 is usually discussed in limited terms since it requires that the FRET channel fluorescence be determined in the absence of the donor. Experiments in cells with these capabilities are usually rare since the absence of a donor precludes energy transfer from occurring. However, the signals measured at the end of a photoswitching cycle of a psFRET experiment closely mimic the condition of imaging the acceptor in the absence of the donor. Thus, the photoswitchable donor gives us an opportunity to directly apply such an equation. Rearranging equation 89 and substituting f_A for f_D, gives the following.

$$Ef_A = \left(\frac{\varepsilon_A\,(\lambda_D^{ex})}{\varepsilon_D\,(\lambda_D^{ex})}\right)\left(\frac{F_{AD}\,(\lambda_A^{em}) - F_A\,(\lambda_A^{em})}{F_A\,(\lambda_A^{em})}\right) \tag{90}$$

Note that $F_{AD}\,(\lambda_A^{em}) - F_A\,(\lambda_A^{em})$ represents direct acceptor excitation subtracted from the FRET channel signal in the absence of the donor and this is the same as $F_{c\,on}$ which we determine as described previously (equation 83). As we also discussed above, we take advantage of the high contrast between the "on" and "off" states of Dronpa to approximate the direct acceptor excitation ($F_A\,(\lambda_A^{em})$) in the absence of the donor using the FRET channel signal after it has been switched off ($I_{DA\,off}$). The relative absorptions of the donor and acceptor are given by $\varepsilon_D\,(\lambda_D^{ex})$ and $\varepsilon_A\,(\lambda_D^{ex})$, respectively. These can be determined from the spectra and extinction coefficients derived from the literature. We determined for excitation at 488 nm that $\varepsilon_D\,(\lambda_D^{ex})$ = 62600 mol^{-1} cm^{-1} and $\varepsilon_A\,(\lambda_D^{ex})$ = 7700 mol^{-1} cm^{-1} for Dronpa and mCherry, respectively. By substituting these known or measured values in equation 90, we can determine the energy transfer and fractional labeling of the acceptor.

$$Ef_A = \left(\frac{\varepsilon_A\,(\lambda_D^{ex})}{\varepsilon_D\,(\lambda_D^{ex})}\right)\left(\frac{F_{c\,on}}{I_{DA\,off}}\right) \tag{91}$$

SI Appendix figure S1. Multi-exponential fits of psFRET data.

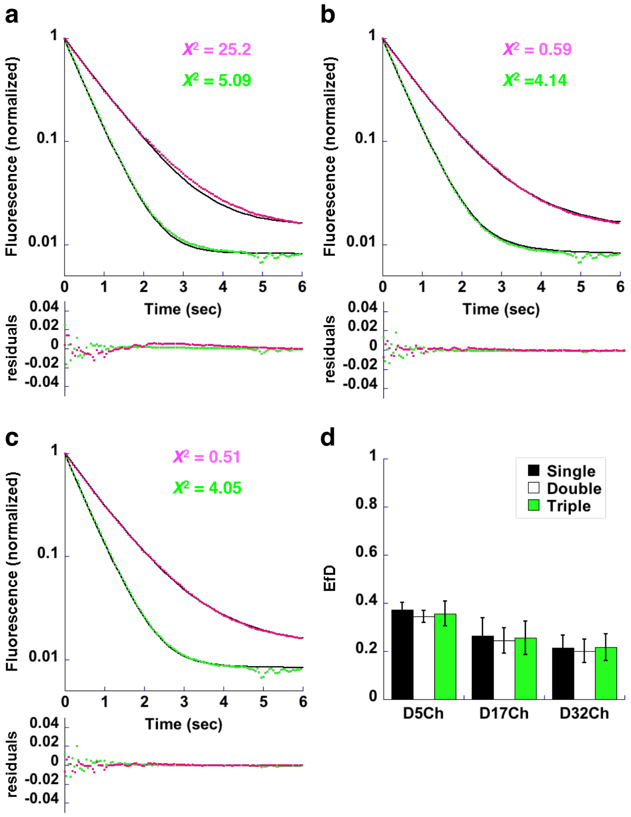

SI Appendix figure S1. Multi-exponential fits of psFRET data. Mean pixel values from regions of interest in Dronpa (green points) expressing cells or D5Ch (magenta) expressing cells were fitted (solid lines) with (**a**) single ($A + B*e^{-k*t}$), (**b**) double ($A + B*e^{-k1*t} + C*e^{-k2*t}$), or (**c**) triple ($A + B*e^{-k1*t} + C*e^{-k2*t} + D*e^{-k3*t}$) exponential decays with offsets. The residuals plots are shown below the data and fit plots. The reduced Chi-square values for these experiments are indicated on the graphs. (**d**) Using the single exponential rate constants or the weighted average rate constants from the double and triple fits of Dronpa alone (k_{Doff}) or from D5Ch, D17Ch, and D32Ch tandem dimers (k_{DAoff}), the FRET efficiencies were determined from $Ef_D = 1 - \frac{k_{DAoff}}{k_{Doff}}$. Data represent mean ± sd (n ≥ 27). For the multi-exponential fits, weighted average rate constants were determined and substituted for k_{Doff} or k_{DAoff} as appropriate. The weighted average rate constants for double exponential fits were calculated using $\frac{B*k_1+C*k_2}{B+C}$ and triple exponential fits were calculated using $\frac{B*k_1+C*k_2+D*k_3}{B+C+D}$. Although better fits could be obtained by increasing the number of terms, the multi-exponential fits resulted in FRET efficiencies similar to the single exponential fits. The reduced Chi-square range for Dronpa alone fits was 0.84-18.9 (single), 0.18-17.3 (double), and 0.15-16.3 (triple). The Chi-square value range for D5Ch fits were 2.66-45.5 (single), 0.24-2.11 (double), and 0.24-1.52 (triple). The Chi-square value range for D17Ch fits were 5.1-62.3 (single), 0.31-5.03 (double), and 0.12-2.88 (triple). The Chi-square value range for D32Ch fits were 5.25-22.6 (single), 0.36-1.78 (double), and 0.23-1.37(triple).

SI Appendix figure S2. Screenshots showing example fits and residuals in psFRET analyses.

a

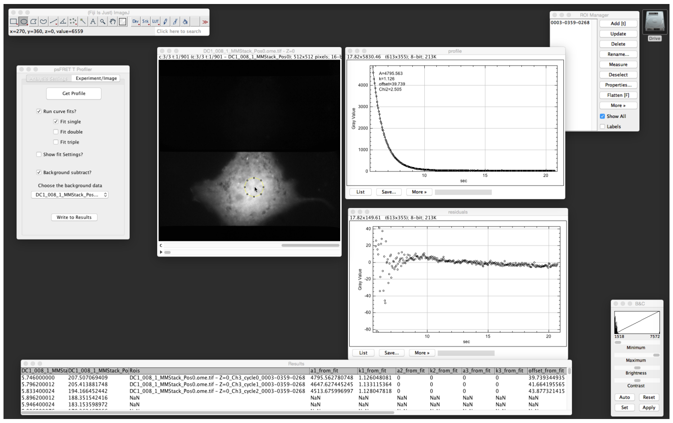

b

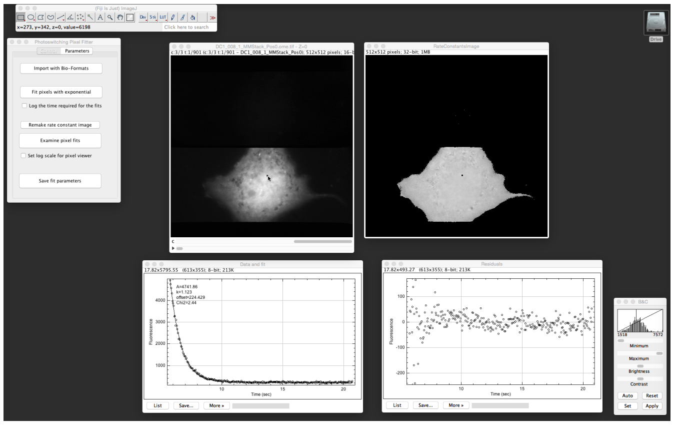

SI Appendix figure S2. Screenshots showing example fits and residuals in psFRET analyses. We have developed ImageJ plugins for analyses of psFRET data. The plugins use Bio-Formats to extract the timestamp information automatically, otherwise user supplied time intervals are requested. These screenshots show these in use with example data and fit plots along with residuals plots. **a.** The psFRET_T_Profiler allows analyses of the mean pixel values in specified regions of interest over time. The fluorescence decays can be to single, double, or triple exponential equations (although we suggest limiting the fits to single or double). The data, fit, and the fit parameters are displayed in one plot and the residuals are displayed in a separate plot window. The data and fit parameters can be written to the results table for each ROI. The results table can be saved and opened in other software, such as Excel, for further analysis and calculation of FRET efficiencies. **b.** The Photoswitching_Pixel_Fitter plugin extracts intensity values at each pixel over a photoswitching experiment and fits that data to a single exponential with offset equation. The final output are images containing the initial signal (Azero), the rate constant, the offset, and the Chi-square at each pixel. The plugin also offers the capability to examine the pixel fits by displaying the data for a selected pixel, the fitted function for selected pixel, and the fit parameters in one plot window. A second window shows the residuals of the fit.

SI Appendix figure S3. Chi-square images of psFRET pixel-by-pixel analysis.

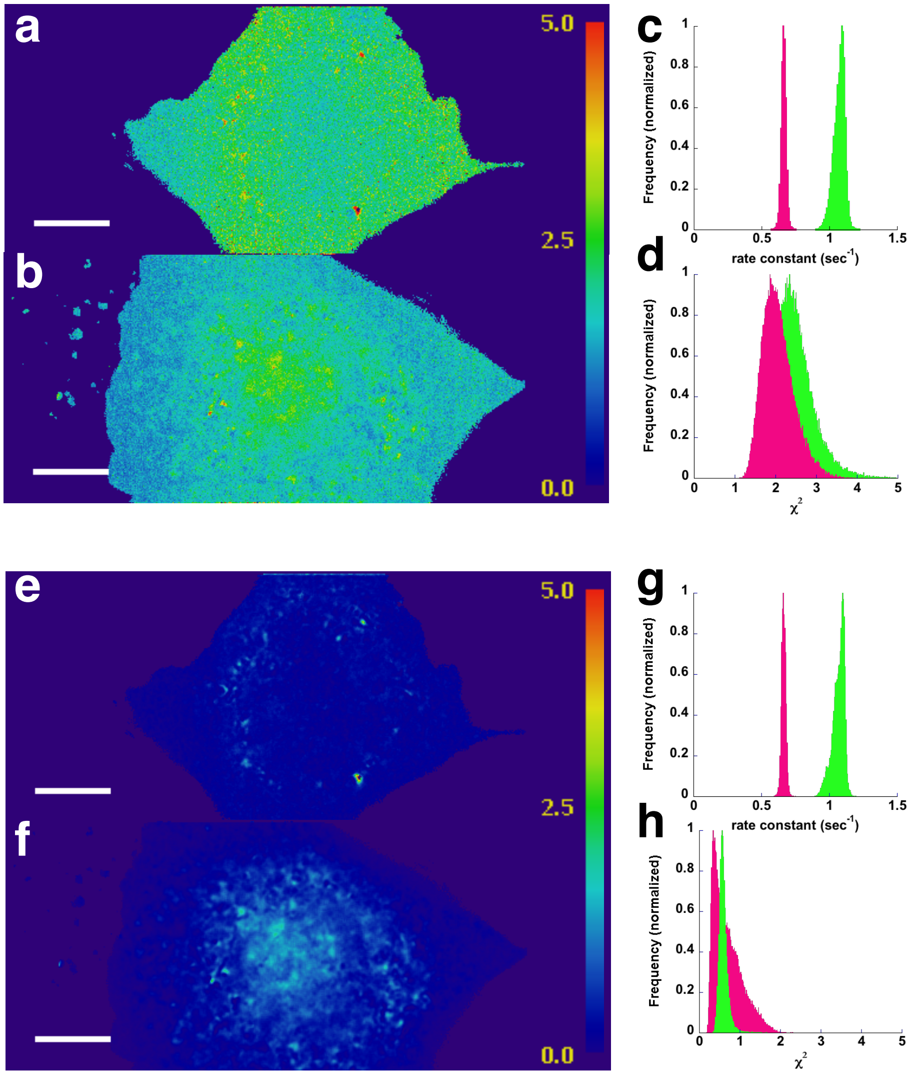

SI Appendix figure S3. Chi-square images of psFRET pixel-by-pixel analysis.
COS7 cells expressing Dronpa or D5Ch were subjected to Dronpa photoswitching and analysis using the Photoswitching_Pixel_Fitter plugin. The Chi-square value for the fit at each pixel was used to create new images of Dronpa (**a**) and D5Ch (**b**). **c.** The rate constants (sec^{-1}) for Dronpa (green) and D5Ch (magenta) are displayed as histograms to show the distributions. **d.** The reduced Chi-square values for Dronpa (green) and D5Ch (magenta) are displayed as histograms to show the distributions. The psFRET images were processed using a 1 pixel mean filter and then analyzed using the plugin. Reduced Chi-square values for the fits at each pixel were used to create new images of Dronpa (**e**) and D5Ch (**f**). The rate constants (sec^{-1}) (**g**) and reduced Chi-square (**h**) for Dronpa (green) and D5Ch (magenta) are displayed as histograms to show the distributions. Scale bars = 10 µm.

SI Appendix figure S4. Uniformity of the illumination is an important technical consideration for the psFRET technique.

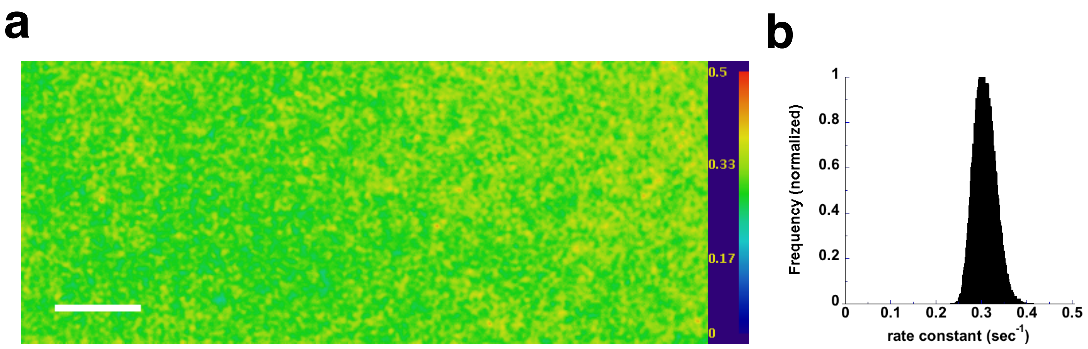

SI Appendix figure S4. Uniformity of the illumination is an important technical consideration for the psFRET technique. We incubated purified Dronpa (~65μM) on a coverslip and washed with 1XPBS. Some protein remained associated with the coverslip, which we then imaged by psFRET and analyzed the data using our pixel fitting plugin. The distribution of the protein is not homogeneous, but it provided a full imaging field of sample to determine and generate a rate constant image (**a**). The lookup table at the right can be used to interpret rate constant (sec^{-1}) values. The values are displayed as a histogram (**b**) to show their distribution. Scale bar = 40 μm.

SI Appendix figure S5. Relative mCherry and Dronpa fluorescence in the test chimeras.

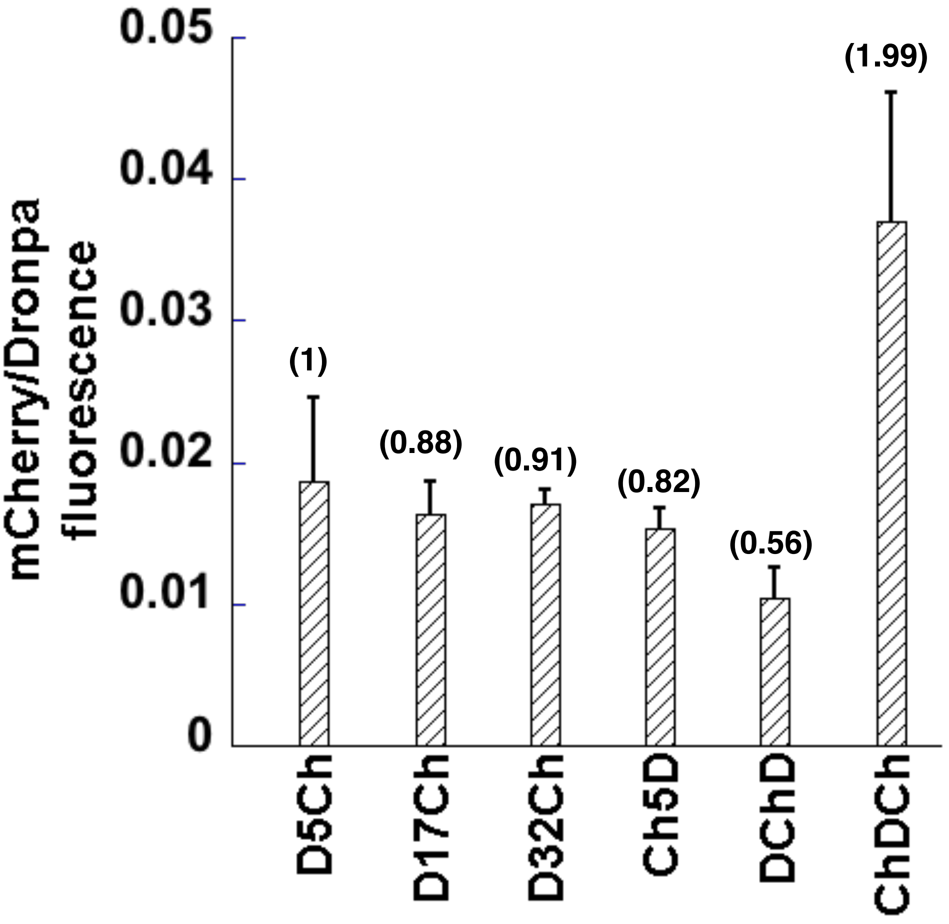

SI Appendix figure S5. Relative mCherry and Dronpa fluorescence in the test chimeras. Dronpa and mCherry fluorescence signals were determined under 488nm excitation. The Dronpa signal was determined in the green channel from the first image of the photoswitching experiment and normalized for the loss in signal due to energy transfer as determined from the photoswitching kinetics. The mCherry signal was determined from the red channel of the last image of the photoswitching cycle. The mCherry/Dronpa ratio was determined by dividing the mCherry fluorescence signal by the Dronpa fluorescence signal. Using D5Ch as the expected signal for a 1:1 Dronpa-mCherry pairing, we can determine the relative fluorescence ratios, which are indicated in parentheses. Data represent mean ± sd ($n \geq 9$).

SI Appendix figure S6. H2B-Dronpa and H2B-mCherry incorporate into chromatin.

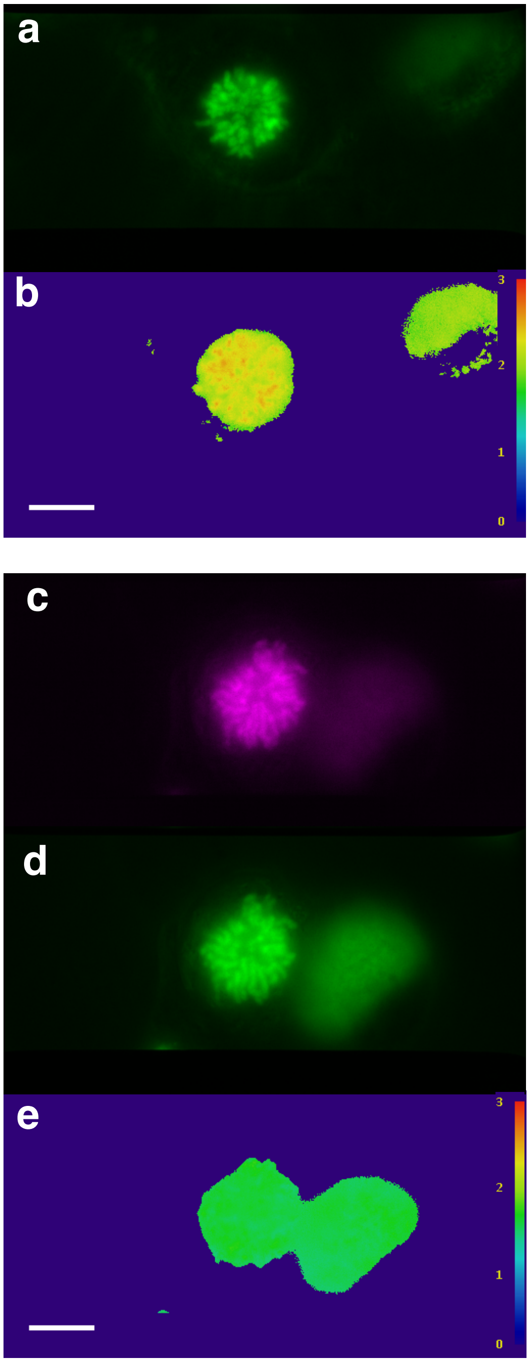

SI Appendix figure S6. H2B-Dronpa and H2B-mCherry incorporate into chromatin. a. Example of an H2B-Dronpa expressing cell in mitosis. **b.** Rate constant image derived from psFRET image analysis of the cell in **a.** Example of a cells expressing H2B-mCherry (**c**) and H2B-Dronpa (**d**) in mitosis. **e.** Rate constant image (sec^{-1}) derived from psFRET image analysis the cells shown in **d.** Scale bars = 10 µm.

SI Appendix figure S7. D5Ch FRET efficiency dependence on the ratio of H2B-mCherry/H2B-Dronpa expression level.

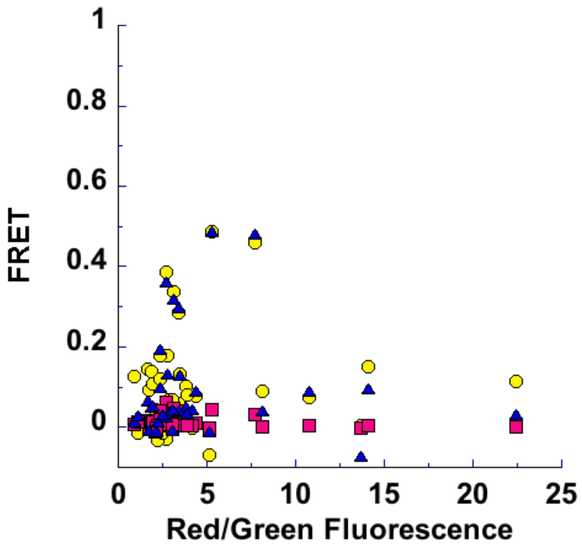

SI Appendix figure S7. FRET efficiency dependence on the ratio of H2B-mCherry/H2B-Dronpa expression level. FRET efficiencies of nuclear regions of interest were determined by fitting photoswitching kinetics (***Ef_D***, ○), from sensitized emission using equation 84 (***Ef_D***, ▲), and sensitized emission using equation 91 (***Ef_A***, □) and are displayed as a function of the ratio of mCherry/Dronpa fluorescence signals.

SI Appendix figure S8. D5Ch FRET efficiency measured in the presence of overexpressed donor or overexpressed acceptor.

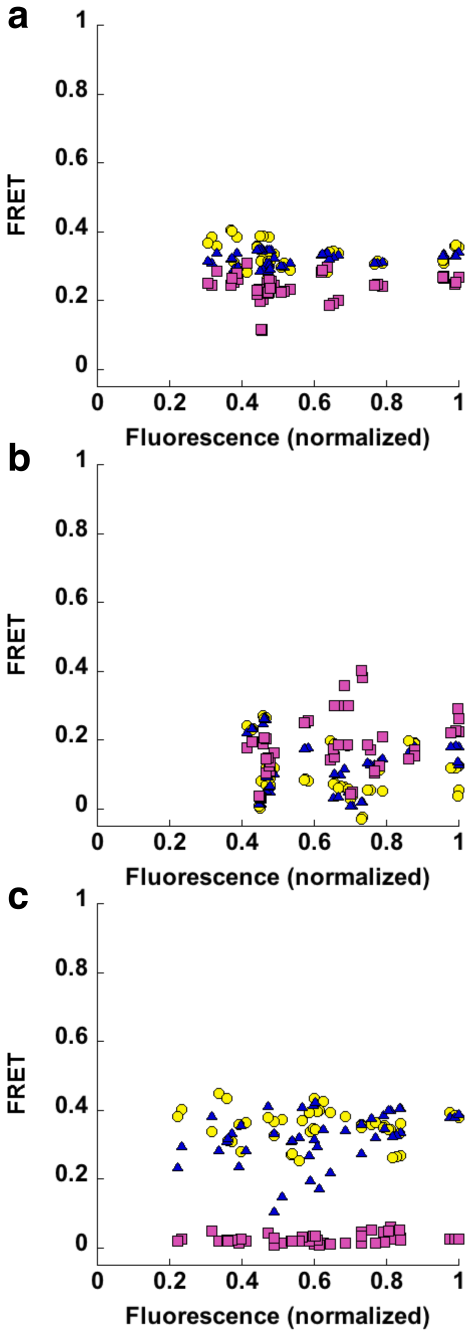

SI Appendix figure S8. D5Ch FRET efficiency measured in the presence of overexpressed donor or overexpressed acceptor. FRET efficiencies were determined by fitting photoswitching kinetics (***Ef$_D$***, ○), from sensitized emission using equation 84 (***Ef$_D$***, ▲), and sensitized emission using equation 91 (***Ef$_A$***, ■) for (**a**) D5Ch, (**b**) D5Ch plus overexpressed Dronpa, and (**c**) D5Ch plus overexpressed mCherry. These are displayed as a function of the total fluorescence signal collected for both channels.

SI Appendix figure S9. A caspase-3 biosensor based on Dronpa-DEVD-mCherry photoswitching rate constants displays heterogeneous responses.

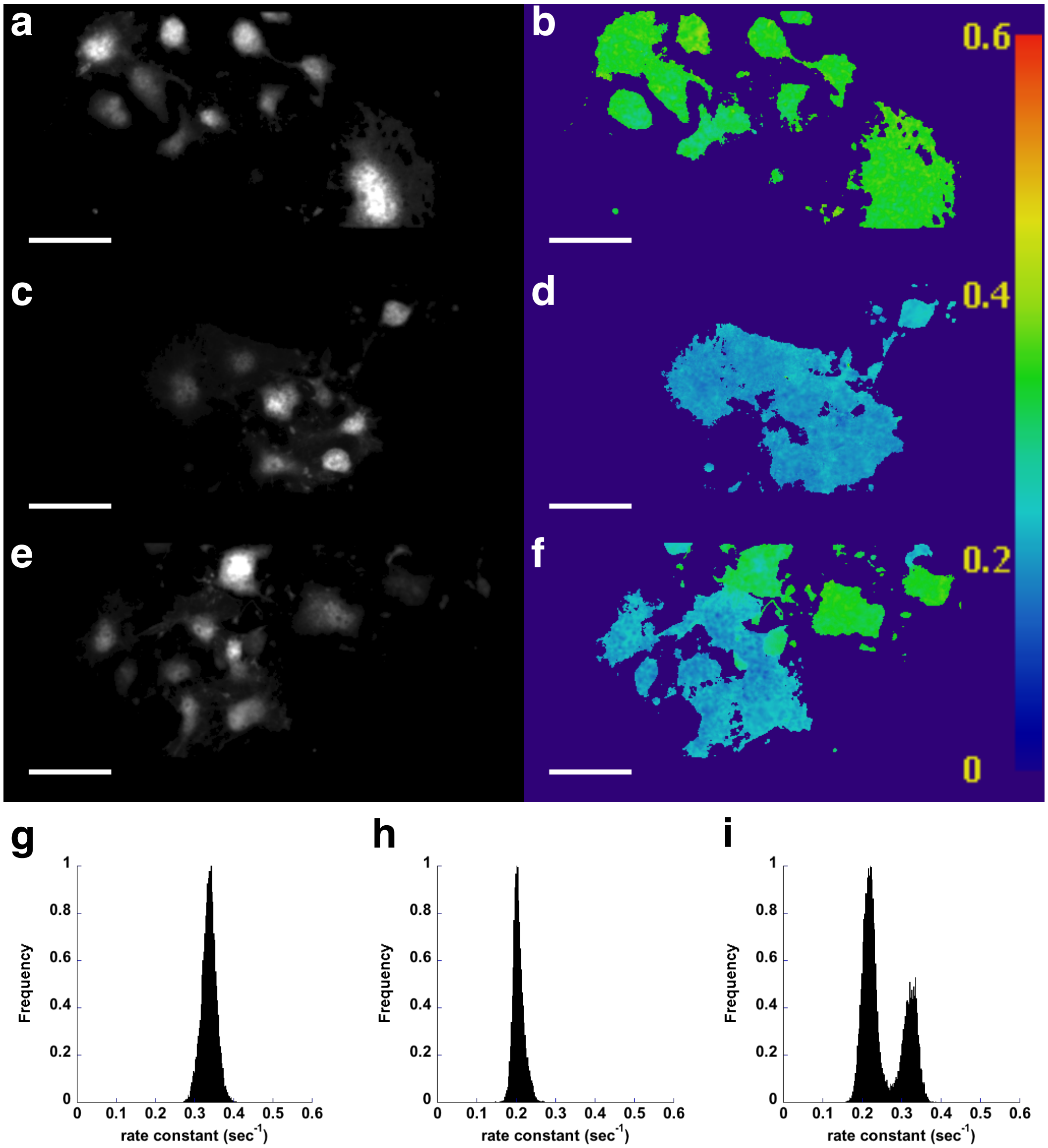

SI Appendix figure S9. A caspase-3 biosensor based on Dronpa-DEVD-mCherry photoswitching rate constants displays heterogeneous responses. **a.** A field of COS 7 cells expressing Dronpa was treated with 2μM staurosporine as described in the main text, imaged using our psFRET protocol, and the corresponding (**b**) rate constant image (sec^{-1}) was generated. **c.** A field of COS 7 cells expressing D5Ch was treated with 2μM staurosporine, imaged using our psFRET protocol, and the corresponding (**d**) rate constant image (sec^{-1}) was generated. **e.** A field of COS 7 cells expressing D-DEVD-Ch was treated with 2μM staurosporine, imaged using our psFRET protocol, and the corresponding (**f**) rate constant image (sec^{-1}) was generated. The rate constants in **b**, **d**, and **f** are displayed as histograms in **g**, **h**, and **i**, respectively. Scale bars = 50 μm.

SI Appendix figure S10. The Dronpa photoswitching rate constant is dependent on cycle number.

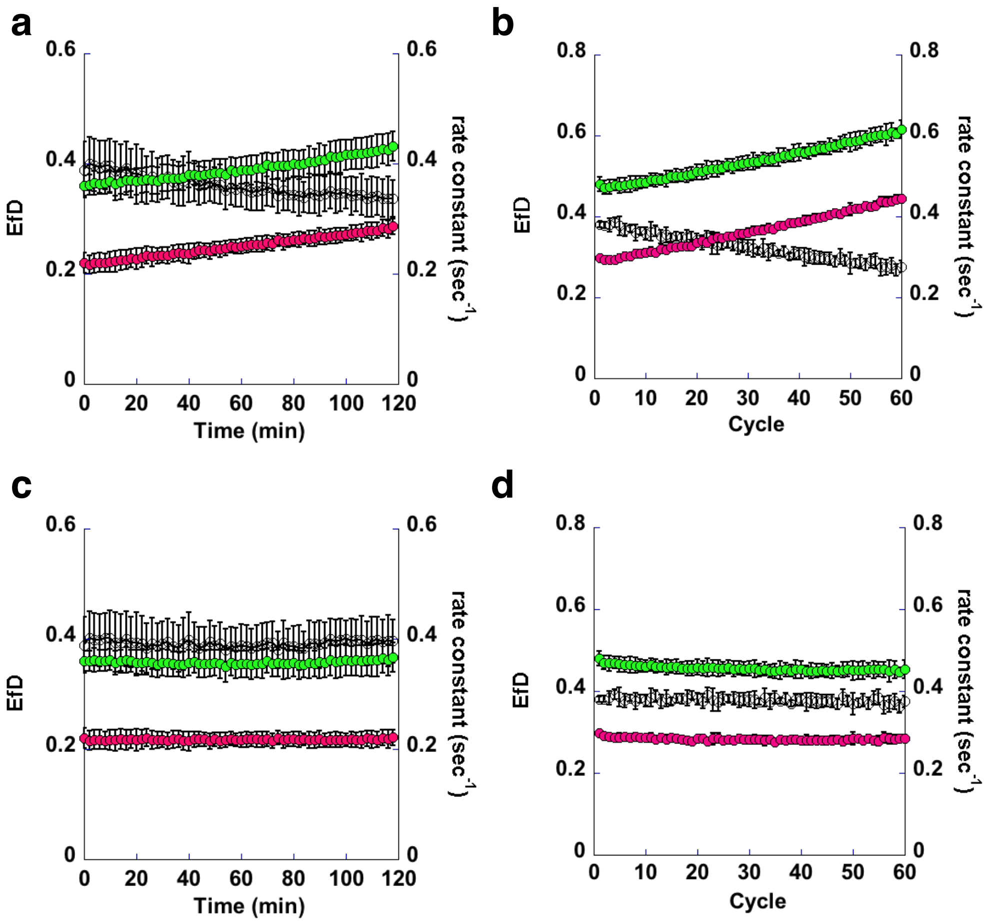

SI Appendix figure S10. The Dronpa photoswitching rate constant is dependent on cycle number. **a.** COS 7 cells expressing Dronpa (filled green circles) or D5Ch (filled magenta circles) and treated with 2 μM staurosporine were imaged using our psFRET protocol over 60 cycles and the photoswitching rates constants determined for each cycle. The FRET efficiency (***Ef$_D$***, open circles) was determined for each cycle. **b**. Untreated COS 7 cells expressing Dronpa (filled green circles) or D5Ch (filled magenta circles) were imaged as in **a**. **c.** The slope of a linear fit of the Dronpa alone photoswitching rate constants in **a** was used to correct for the change in Dronpa photoswitching. The slope was multiplied by the cycle number and subtracted from the rate constants for both the Dronpa control (filled green circles) and D5Ch (filled magenta circles). The FRET efficiency (***Ef$_D$***, open circles) was then determined for each cycle using the corrected rate constants. **d.** The slope of a linear fit of the Dronpa alone photoswitching rate constants in **b** was used to correct for the change in Dronpa photoswitching in both the Dronpa control (filled green circles) and D5Ch (filled magenta circles). This correction was also applied to the D-DEVD-Ch data in figure 6c of the main text. Data represent mean ± sd (n = 8 for **a**,**c** and n = 10 for **b**,**d**).

SI Appendix figure S11. Dronpa and mCherry photostability during psFRET photoswitching cycles.

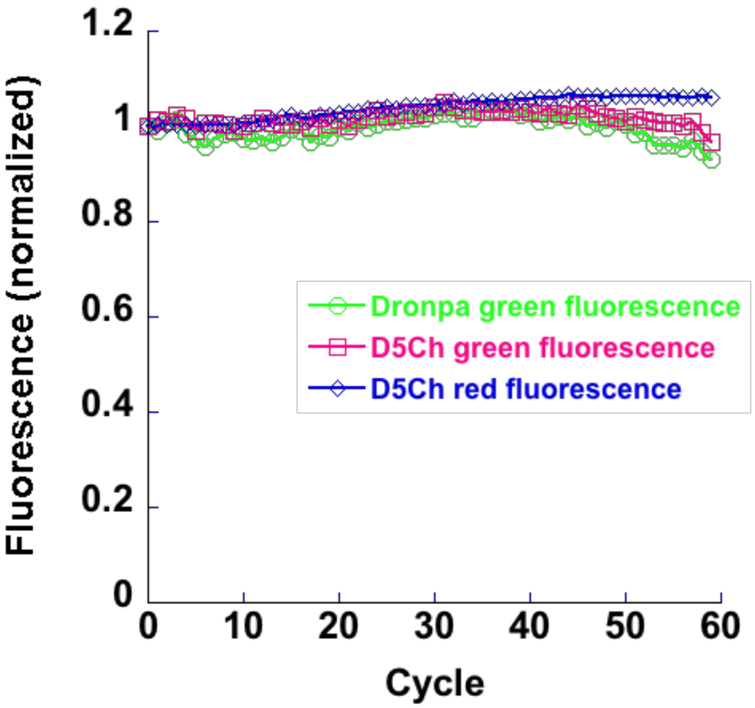

SI Appendix figure S11. Dronpa and mCherry photostability during psFRET photoswitching cycles. The COS 7 cells expressing Dronpa (green circles) or D5Ch (magenta squares and blue diamonds) imaged for figure 6c (main text) and SI Appendix figure 6 were used to assess the extent of photobleaching in these experiments. The green fluorescence signals (Dronpa green fluorescence and D5Ch green fluorescence) measured in the first image immediately after photoswitching "on" with 405 nm light were averaged (n = 8 and n = 10 respectively) and normalized to the initial intensity. In our psFRET protocol, we collect a 568nm excited red fluorescence image at each cycle prior to photoswitching "on" with 405 nm light. The mean intensities of the cells in these images were averaged (n = 10) and normalized to the initial value (D5Ch red fluorescence).

SI Appendix figure S12. Dronpa photoswitching rate constant fusion protein independence.

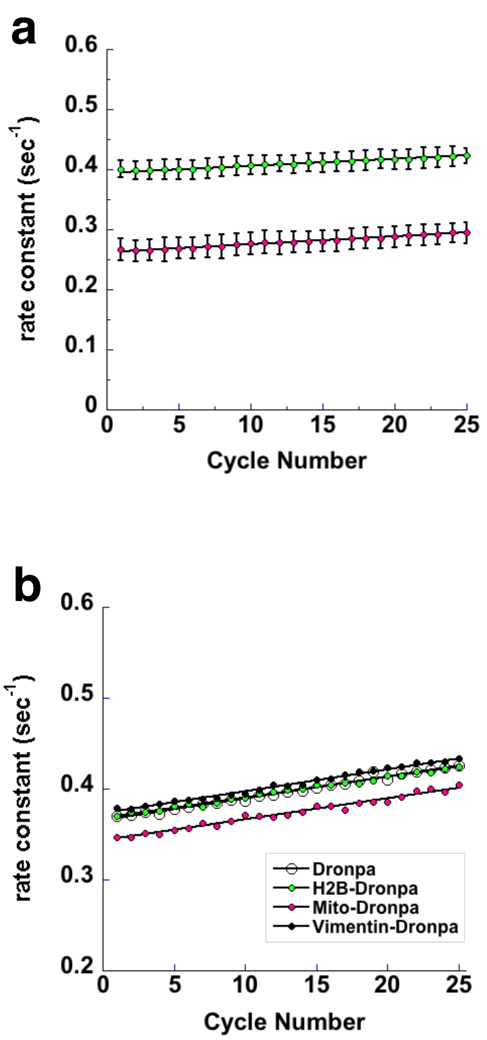

SI Appendix figure S12. Dronpa photoswitching rate constant fusion protein independence. a. COS 7 cells expressing Dronpa (green) or Ch5D (magenta) were imaged using our psFRET protocol and the photoswitching rate constants determined for each cycle. Linear fits of these data show similar slopes. Data represent mean ± sd (n = 13). **b**. COS 7 cells expressing Dronpa, H2B-Dronpa, Mito-Dronpa, or Vimentin-Dronpa were imaged using our psFRET protocol and the photoswitching rate constants determined for each cycle. Linear fits of these data indicate similar slopes. Data represent the means. Error bars are omitted for clarity. The standard deviations ranged from 0.015 - 0.032. (n ≥ 19 for each).

SI Appendix figure S13. Photoswitching rate constant cycle dependence in Dronpa and other photoswitchable fluorescent proteins.

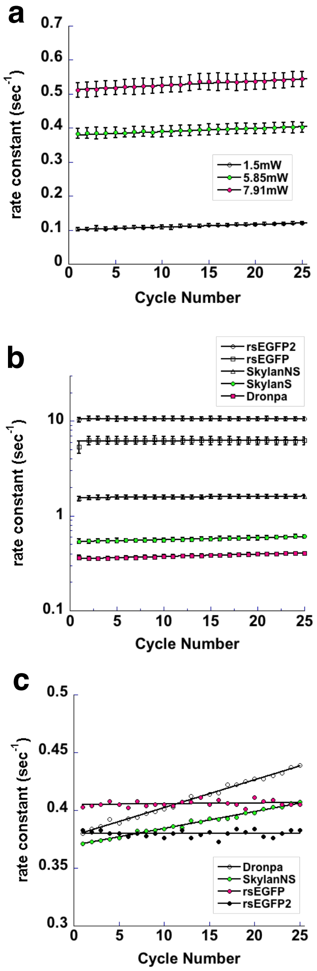

SI Appendix figure S13. Photoswitching rate constant cycle dependence in Dronpa and other photoswitchable fluorescent proteins. a. COS 7 cells expressing Dronpa were imaged at three power levels (indicated on the plot) using our psFRET protocol and the photoswitching rate constants determined for each cycle. Linear fits of these data indicate that all three have similar slopes. Data represent mean ± sd (1.5mW for n = 17, n = 19 for 5.85mW, and n = 16 for 7.91mW). **b.** COS 7 cells expressing rsEGFP2, rsEGFP, SkylanNS, SkylanS, or Dronpa were imaged at the same illumination power using our psFRET protocol and the photoswitching rate constants determined for each cycle. Linear fits of these data show slightly positive slopes ranging from ~0.001 to ~0.0035 per cycle. Data represent mean ± sd (n = 18 for each protein). **c.** COS 7 cells expressing Dronpa, SkylanNS, rsEGFP, or rsEGFP2 were imaged using our psFRET protocol using illumination powers to closely match their photoswitching rate constants. The photoswitching rate constants were determined for each cycle followed by linear fits of these data. Data represent the means. Error bars are omitted for clarity. The standard deviations ranged from 0.012 - 0.026. (n ≥ 16 for each).

SI Appendix figure S14. H2B-Dronpa modeled in a nucleosome.

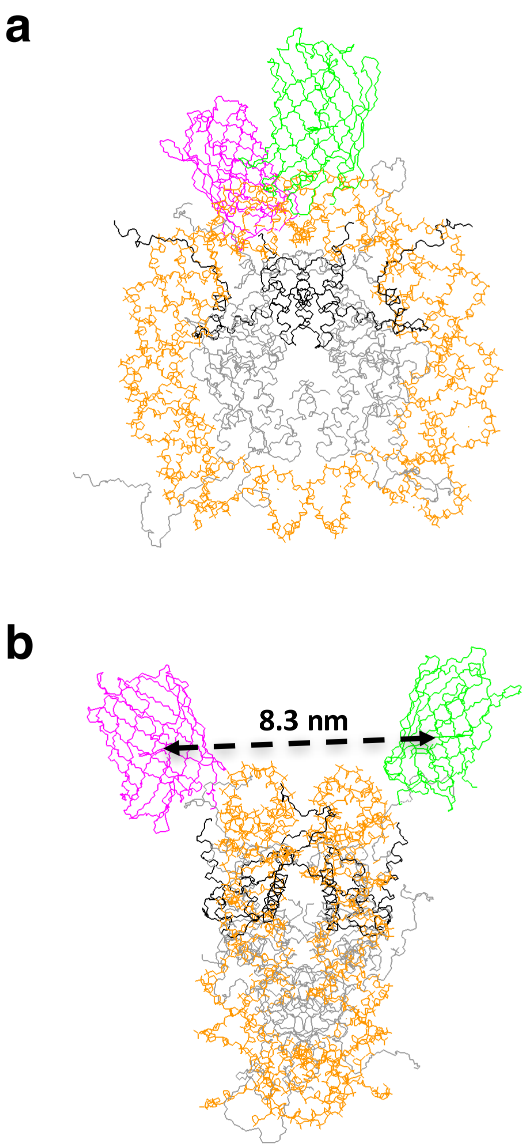

SI Appendix figure S14. H2B-Dronpa modeled in a nucleosome. A nucleosome is show in conjunction with two Dronpa molecules to show the probable locations of H2B-Dronpa and H2B-mCherry. These images show the DNA (orange), histones H2A, H3, H4 (light gray), and H2B (black). The N termini of two Dronpa molecules (green and magenta) were positioned in close proximity to the C termini of the two H2B molecules. **a.** The nucleosome is displayed from a top-down view showing the core histone proteins (gray) with the DNA (orange) encircling this structure. **b.** The nucleosome is shown from the side (orthogonal to the view in a). The distance between the fluorescent protein chromophores was estimated using the distance tool of the Swiss PDB Viewer. Images were produced using the Swiss PDB Viewer using the nucleosome structure pdb file 1eqz and the Dronpa structure pdb file 2IE2.

SI Appendix table S1. Example processing time for the Photoswitching_Pixel_Fitter plugin.

Computer	Processing time (512 x 512 x 300 frames)	
	**Non-threaded**	**Threaded**
**MacBook Pro, 2.66 GHz Intel Core i7, 2 cores**	348 sec	189 sec
**Mac Pro, 2 x 2.26 GHz Quad Core Intel Xeon, 8 cores**	399 sec	58 sec

Oligonucleotide sequences and cloning strategies

The Dronpa-5-mCherry (D5Ch) plasmid was constructed by PCR amplifying mCherry (from pmCherry-C1) without its first methionine (ATG) using the N-terminal annealing primer 5'-GATCAGATCTGTGAGCAAGGGCGAGGAG-3' containing a BglII site (underlined) and the C-terminal annealing primer 5'-GATCGAATTCCTTGTACAGCTCGTC-3' containing an EcoRI site (underlined) as previously described[13]. Both the mCherry PCR product and pDronpa-C1 were digested with BglII and EcoRI then ligated together creating the five-amino acid linker SGLRS.

The Dronpa-17-mCherry (D17Ch) plasmid was constructed by PCR amplifying mCherry (from pmCherry-C1) without its first two amino acids methionine and valine (ATG and GTG) as previously described[13] using the N-terminal annealing primer 5'-GATCGGTACCAGCAAGGGCGAGGAGGAT-3' containing a KpnI site (underlined) and the C-terminal annealing primer 5'-GATCGGATCCTTACTTGTACAGCTCGTCCAT-3'containing a BamHI site (underlined). Both the mCherry PCR product and pDronpa-C1 were digested with KpnI and BamHI then ligated together creating the seventeen-amino acid linker SGLRSRAQASNSAVDGT.

The Dronpa-32-mCherry (D32Ch) plasmid was constructed as previously described[13] by replacing the NheI (underlined) to AgeI (underlined) sequence in pmCherry-N1 with the synthesized oligonucleotide 5'-GCTAGCGCCACCATGGATCCTACTAGTGGCCTCGAGACGCGTGATATCAGATCTGAGAACCTGTACTTCCAGGGCCCGCGGGAATTCCCCGGGGGTACCGCTGGACCGGT-3' by digestion with NheI and AgeI of both insert and pmCherry-N1 followed by ligation. Dronpa from pDronpa-C1 was then PCR amplified without its first methionine (ATG) or a stop codon (TAA) using the N-terminal annealing primer 5'-GATCGGATCCTAGTGTGATTAAACCAGACATG-3' containing a BamHI site (underlined) and the C-terminal annealing primer 5'-GATCACTAGTCTTGGCCTGCCTCGGCAGCTC-3' containing a SpeI site (underlined). Both the Dronpa PCR product and newly made pmCherry-N1 were digested with BamHI and SpeI then ligated together to create Dronpa with an ATG start codon just prior to the BamHI site followed by the 32-amino acid linker, TSGLETRDIRSENLYFQGPREFPGGTAGPVAT, and mCherry.

The mCherry-5-Dronpa (Ch5D) plasmid was constructed in a similar manner as D5Ch described above. Dronpa (from pDronpa-C1) was PCR amplified without its first methionine (ATG) using the N-terminal annealing primer 5'-GATCAGATCTAGTGTGATTAAACCAGAC-3' containing a BglII site (underlined) and the C-terminal annealing primer 5'-GATCGAATTCCTTGGCCTGCCTCGGCAG-3' containing an EcoRI site (underlined). Both the Dronpa PCR product and pmCherry-C1 were digested with BglII and EcoRI then ligated together creating the five-amino acid linker SGLRS.

The mCherry-Dronpa-mCherry (ChDCh) plasmid was constructed by PCR amplifying mCherry (from pmCherry-C1) without its first methionine (ATG) using the N-terminal annealing primer 5'-GATCGTCGACGGGTGAGCAAGGGCGAGGAG-3' containing a SalI site (underlined) and the C-terminal annealing primer 5'-GATCGGATCCCTTGTACAGCTCGTCCAT-3' containing a BamHI site (underlined). Both the mCherry PCR product and pCh5D (described above) were digested with SalI and BamHI then ligated together creating the six-amino acid linker EFCSRR between Dronpa and C-Terminal mCherry.

The Dronpa-mCherry-Dronpa (DChD) plasmid was constructed by PCR amplifying Dronpa (from pDronpa-C1) without its first methionine (ATG) using the N-terminal annealing primer 5'-GATCGTCGACGGAGTGTGATTAAACCAGAC-3' containing a SalI site (underlined) and the C-terminal annealing primer 5'-GATCGGATCCCTTGGCCTGCCTCGGCAG-3' containing a BamHI site (underlined). Both the Dronpa PCR product and pD5Ch (described above) were digested with SalI and BamHI then ligated together creating the six-amino acid linker EFCSRR between mCherry and the C-terminal Dronpa.

The Dronpa-mCherryAmber (D5ChA) plasmid was constructed by first making the point mutation Y67C (underlined) in mCherry (from pmCherry-C1) via PCR as previously described[13] using the oligonucleotide primer 5'-TCCCCTCAGTTCATGTGCGGCTCCAAGGCCTAC-3' and its reverse complement 5'-GTAGGCCTTGGAGCCGCACATGAACTGAGGGGA-3'. The same N-terminal annealing primer containing BglII and C-terminal annealing primer containing EcoRI described above to make D5Ch were used to PCR amplify newly made mCherry-Y67C which we refer to as mCherryAmber. Both the mCherryAmber PCR product and pDronpa-C1 were digested with BglII and EcoRI then ligated together creating the five-amino acid linker SGLRS.

The Dronpa-DEVD-mCherry plasmid was constructed as follows. The oligonucleotide primer containing the linker and DEVD sequence 5'-GATCTCCGGACTCGGCGGTACCGGCAGTGGCAGCGGAGATGAGGTCGATGGTAGATCTGATC-3' and its reverse complement 5'-GATCAGATCTACCATCGACCTCATCTCCGCTGCCACTGCCGGTACCGCCGAGTCCGGAGATC-3' described previously[14] were designed and synthesized (Eurofins Genomics, Louisville, KY) with BspEI and BglII endonuclease restriction sites (underlined). These oligonucleotides were annealed and digested with BspEI and BglII (New England Biolabs, Inc.) and ligated into a similarly digested D5Ch plasmid described above.

The Dronpa-5-Ultramarine and Dronpa-DEVD-Ultramarine plasmids were constructed as follows. The cDNA sequence for Ultramarine[15] was obtained from Mark Prescott and synthesized (Eurofins Genomics, Louisville, KY). The Ultramarine encoding sequence was PCR amplified using oligonucleotides designed to anneal to the N-terminal encoding sequence 5'-ATCAGATCTATGGCTAGCGTGATCGC-3' and C-terminal encoding sequence 5'-GATCCTGCAGAATTCGGCGACCACAGGTTTGC-3' (Eurofins Genomics) with the restriction endonuclease sites BglII and PstI (underlined), respectively. This fragment was digested with BglII and PstI (New England Biolabs, Inc.) and ligated into a similarly digested Dronpa-5-mCherry plasmid described above to replace the mCherry with Ultramarine.

The H2B-mCherry and H2B-mPlum were gifts obtained from the collection of Michael Davidson. The H2B-Dronpa was constructed by digesting the H2B-mPlum with NheI and BamHI to remove a fragment encoding the H2B cDNA sequence. This fragment was purified and subsequently ligated into a similarly digested Dronpa-N1 plasmid. The construction of the Dronpa-N1 plasmid was described previously[16].

A plasmid encoding mito-PAGFP[17] was a gift from Richard Youle (National Institutes of Health). Mito-PAGFP was digested with NheI and BamHI to isolate a fragment containing the cDNA for the cytochrome c oxidase mitochondria targeting sequence. This was ligated into a similarly digested Dronpa-N1 plasmid to produce Mito-Dronpa.

A plasmid encoding Vimentin-PSmOrange[18] was the gift of Vlad Verkhusha (Albert Einstein College of Medicine). It was digested with NheI and BamHI to isolate a fragment containing the cDNA for vimentin. This was ligated into a similarly digested Dronpa-N1 plasmid to produce Vimentin-Dronpa.

A bacteria expression plasmid, pQE31-rsEGFP[19], was a gift from Stefan Jakobs and Stefan Hell (Max Plank Institute for Biophysical Chemistry). pQE31-rsEGFP2[20] was constructed using QuickChange mutagenesis using pQE31-rsEGFP as the template and mutagenesis primers for T65A (5'-CCACCCTGGCCTACGGCGTG-3'; 5'-CACGCCGTAGGCCAGGGTGG-3'), A150V (5'-GCCACAACGTCTATATCATGG-3'; 5'-CCATGATATAGACGTTGTGGC-3'), and N205S (5'-GCACCCAGTCCAAGCTGAGC-3'; 5'-GCTCAGCTTGGACTGGGTGC-3') (Eurofins Genomics). Mammalian expression plasmids were constructed by amplifying the cDNA sequences for rsEGFP and rsEGFP2 from pQE31-rsEGFP and pQE31-rsEGFP2, respectively, using primers designed to anneal to the N-terminal encoding sequence 5'-GATCGGATCCACCGGTCGCCACCATGGTGAGCAAGGGC -3' and C-terminal encoding sequence 5'-GATCCGCGGCCGCTTTACTTGTACAGCTC -3' (Eurofins Genomics) with the restriction endonuclease sites AgeI and BsrGI (underlined), respectively. These fragments were digested with AgeI and BsrGI (New England Biolabs, Inc.) and ligated into similarly digested PAmCherry-N1 and PAmCherry-C1 plasmids[21] replacing PAmCherry with rsEGFP or rsEGFP2 to produce rsEGFP-N1, rsEGFP-C1, rsEGFP2-N1, and rsEGFP2-C1.

The SkylanS and SkylanNS mammalian expression plasmids were developed as follows. A bacteria expression plasmid, pRSETA-mEos2[22], was a gift from Loren Looger (Janelia Research Campus). The mammalian expression plasmid, mEos-N1 was constructed by amplifying the cDNA sequence from pRSETA-mEos2 using primers designed to anneal to the N-terminal encoding sequence 5'-GATCGGATCCACCGGTCGCCACCATGAGTGCGATTAAG-3' and C-terminal encoding sequence 5'-GATCCGCGGCCGCTTTATCGTCTGGCATT-3' (Eurofins Genomics) with the restriction endonuclease sites BamHI and NotI (underlined), respectively. These fragments were digested with BamHI and NotI (New England Biolabs, Inc.) and ligated into similarly digested pEGFP-N1 replacing EGFP with mEos2. The mammalian expression plasmid, mEos-C1 was constructed by amplifying the cDNA sequence from pRSETA-mEos2 using primers designed to anneal to the N-terminal encoding sequence 5'-GATCACCGGTCGCCACCATGAGTGCGATTAAG-3' and C-terminal encoding sequence 5'-GATCCTCGAGATCTGAGTCCGGATCGTCTGGCATTGTC-3' (Eurofins Genomics) with the restriction endonuclease sites AgeI and XhoI (underlined), respectively. These fragments were digested with AgeI and XhoI (New England Biolabs, Inc.) and ligated into similarly digested pEGFP-C1 replacing EGFP with mEos2. The mEos2 plasmids were used as templates and mutated to mEos3.1 and mEos3.2[23] using QuickChange PCR to make mEos3.2-N1 and mEos3.2-C1 plasmids. The mutagenesis primers were I102N (5'-GGGGGCATTTGCAATGCCAGAAACGAC-3'; 5'-GTCGTTTCTGGCATTGCAAATGCCCCC-3'), H158E (5'-ACGGGTGATATTGAGATGGCTTTGTTG-3'; 5'-CAACAAAGCCATCTCAATATCACCCGT-3'), and Y189A (5'-AAGTTACCAGGCGCCCACTTTGTGGAC-3'; 5'-GTCCACAAAGTGGGCGCCTGGTAACTT-3') (Eurofins Genomics). The mEos3.2 plasmids were used as templates and mutated using QuickChange PCR to make mEos3.1-N1 and mEos3.1-C1 plasmids. The mutagenesis primers were I157V (5'-CTGACGGGTGATGTTGAGATGGCTTTG-3'; 5'-CAAAGCCATCTCAACATCACCCGTCAG-3') (Eurofins Genomics). The mEos3.1 plasmids were

used as templates and mutated to SkylanS[24] and SkylanNS[25] using QuickChange PCR. To make SkylanS-N1 and SkylanS-C1 plasmids, the mutagenesis primers were H62S (5'-ACCACTGCATTCAGTTACGGCAACAGG-3'; 5'- CCTGTTGCCGTAACTGAATGCAGTGGT-3') (Eurofins Genomics). To make SkylanNS-N1 and SkylanNS-C1 plasmids, the mutagenesis primers were H62L (5'-ACCACTGCATTCTTGTACGGCAACAGG-3'; 5'-CCTGTTGCCGTACAAGAATGCAGTGGT-3') (Eurofins Genomics).

References

1 Kubitscheck, U. *et al.* Fluorescence resonance energy transfer on single living cells. Application to binding of monovalent haptens to cell-bound immunoglobulin E. *Biophys J* **60**, 307-318, doi:10.1016/S0006-3495(91)82055-0 (1991).

2 Kubitscheck, U., Schweitzer-Stenner, R., Arndt-Jovin, D. J., Jovin, T. M. & Pecht, I. Distribution of type I Fc epsilon-receptors on the surface of mast cells probed by fluorescence resonance energy transfer. *Biophys J* **64**, 110-120, doi:10.1016/S0006-3495(93)81345-6 (1993).

3 Young, R. M., Arnette, J. K., Roess, D. A. & Barisas, B. G. Quantitation of fluorescence energy transfer between cell surface proteins via fluorescence donor photobleaching kinetics. *Biophys J* **67**, 881-888, doi:10.1016/S0006-3495(94)80549-1 (1994).

4 Hoppe, A., Christensen, K. & Swanson, J. A. Fluorescence resonance energy transfer-based stoichiometry in living cells. *Biophys J* **83**, 3652-3664, doi:10.1016/S0006-3495(02)75365-4 (2002).

5 Chen, H., Puhl, H. L., 3rd, Koushik, S. V., Vogel, S. S. & Ikeda, S. R. Measurement of FRET efficiency and ratio of donor to acceptor concentration in living cells. *Biophys J* **91**, L39-41, doi:10.1529/biophysj.106.088773 (2006).

6 Gordon, G. W., Berry, G., Liang, X. H., Levine, B. & Herman, B. Quantitative fluorescence resonance energy transfer measurements using fluorescence microscopy. *Biophys J* **74**, 2702-2713, doi:10.1016/S0006-3495(98)77976-7 (1998).

7 Zal, T. & Gascoigne, N. R. Photobleaching-corrected FRET efficiency imaging of live cells. *Biophys J* **86**, 3923-3939, doi:10.1529/biophysj.103.022087 (2004).

8 Vogel, S. S., Thaler, C. & Koushik, S. V. Fanciful FRET. *Sci STKE* **2006**, re2, doi:10.1126/stke.3312006re2 (2006).

9 Piston, D. W. & Kremers, G. J. Fluorescent protein FRET: the good, the bad and the ugly. *Trends Biochem Sci* **32**, 407-414, doi:10.1016/j.tibs.2007.08.003 (2007).

10 Zeug, A., Woehler, A., Neher, E. & Ponimaskin, E. G. Quantitative intensity-based FRET approaches--a comparative snapshot. *Biophys J* **103**, 1821-1827, doi:10.1016/j.bpj.2012.09.031 (2012).

11 Lakowicz, J. R. *Principles of fluorescence spectroscopy*. 2nd edn, (Kluwer Academic/Plenum, 1999).

12 Hoppe, A. D. in *Imaging Cellular and Molecular Biological Functions* (eds S. L. Shorte & F. Frischknecht) Ch. 6, 157-181 (Springer, 2007).

13 Koushik, S. V., Chen, H., Thaler, C., Puhl, H. L., 3rd & Vogel, S. S. Cerulean, Venus, and VenusY67C FRET reference standards. *Biophys J* **91**, L99-L101, doi:10.1529/biophysj.106.096206 (2006).

14 Ai, H. W., Hazelwood, K. L., Davidson, M. W. & Campbell, R. E. Fluorescent protein FRET pairs for ratiometric imaging of dual biosensors. *Nat Methods* **5**, 401-403, doi:10.1038/nmeth.1207 (2008).

15 Pettikiriarachchi, A., Gong, L., Perugini, M. A., Devenish, R. J. & Prescott, M. Ultramarine, a chromoprotein acceptor for Forster resonance energy transfer. *PLoS One* **7**, e41028, doi:10.1371/journal.pone.0041028 (2012).

16 Betzig, E. *et al.* Imaging intracellular fluorescent proteins at nanometer resolution. *Science* **313**, 1642-1645 (2006).
17 Karbowski, M. *et al.* Quantitation of mitochondrial dynamics by photolabeling of individual organelles shows that mitochondrial fusion is blocked during the Bax activation phase of apoptosis. *J Cell Biol* **164**, 493-499, doi:10.1083/jcb.200309082 (2004).
18 Subach, O. M. *et al.* A photoswitchable orange-to-far-red fluorescent protein, PSmOrange. *Nat Methods* **8**, 771-777, doi:nmeth.1664 [pii]
10.1038/nmeth.1664 (2011).
19 Grotjohann, T. *et al.* Diffraction-unlimited all-optical imaging and writing with a photochromic GFP. *Nature* **478**, 204-208, doi:nature10497 [pii]
10.1038/nature10497 (2011).
20 Grotjohann, T. *et al.* rsEGFP2 enables fast RESOLFT nanoscopy of living cells. *Elife* **1**, e00248, doi:10.7554/eLife.00248
00248 [pii] (2012).
21 Subach, F. V. *et al.* Photoactivatable mCherry for high-resolution two-color fluorescence microscopy. *Nat Methods* **6**, 153-159 (2009).
22 McKinney, S. A., Murphy, C. S., Hazelwood, K. L., Davidson, M. W. & Looger, L. L. A bright and photostable photoconvertible fluorescent protein. *Nat Methods* **6**, 131-133 (2009).
23 Zhang, M. *et al.* Rational design of true monomeric and bright photoactivatable fluorescent proteins. *Nat Methods* **9**, 727-729, doi:10.1038/nmeth.2021 (2012).
24 Zhang, X. *et al.* Development of a reversibly switchable fluorescent protein for super-resolution optical fluctuation imaging (SOFI). *ACS Nano* **9**, 2659-2667, doi:10.1021/nn5064387 (2015).
25 Zhang, X. *et al.* Highly photostable, reversibly photoswitchable fluorescent protein with high contrast ratio for live-cell superresolution microscopy. *Proc Natl Acad Sci U S A* **113**, 10364-10369, doi:10.1073/pnas.1611038113 (2016).
